# Supplementary material for: Myeloperoxidase transforms chromatin into neutrophil extracellular traps
Source: Nature. 2025 Sep 17;647(8090):747–56. doi: 10.1038/s41586-025-09523-9 (PMC12629992; doi:10.1038/s41586-025-09523-9)
Supplement: Supplementary file 1 — Supplementary Tables 1 and 2 and Supplementary Figs 1–29. Supplementary Figs. 22–29 contain the uncropped raw blots and gels. [file 41586_2025_9523_MOESM1_ESM.pdf]

nature portfolio

https://doi.org/10.1038/s41586-025-09523-9

Supplementary information

Myeloperoxidase transforms chromatin into neutrophil extracellular traps

In the format provided by the authors and unedited

Supplementary information for

Myeloperoxidase transforms chromatin into Neutrophil Extracellular Traps

Garth Lawrence Burn[1*], Tobias Raisch[2*], Sebastian Tacke[2], Moritz Winkler[1], Daniel Prumbaum[2], Stephanie Thee[3,4], Niclas Gimber[5], Stefan Raunser[2], Arturo Zychlinsky[1]

[1]Department of Cellular Microbiology, Max Planck Institute for Infection Biology, Charitéplatz 1, 10117 Berlin, Germany

[2]Department of Structural Biochemistry, Max Planck Institute of Molecular Physiology, 44227 Dortmund, Germany.

[3]Department of Pediatric Respiratory Medicine, Immunology and Critical Care Medicine, Charité - Universitätsmedizin Berlin, corporate member of Freie Universität Berlin and Humboldt-Universität zu Berlin, Berlin, Germany

[4]Berlin Institute of Health at Charité – Universitätsmedizin Berlin, Berlin, Germany

[5]Advanced Medical Bioimaging Core Facility, Charité-Universitätsmedizin, 10117 Berlin, Germany

*These authors contributed equally

Supplementary Table 1. Cryo-EM SPA datasets and models.

	rMPO/nucl.	Free nucl.	MPO monomer/nucl.	MPO dimer/nucl.	MPO dimer/nucl. (intermediate state)
**PDB entry**	9GEN	9GEO	9GEP	9GEQ	9GER
**EMDB entry**	EMD-51295	EMD-51296	EMD-51297	EMD-51298<br>EMD-51299<br>EMD-51300<br>EMD-51301	EMD-51302<br>EMD-51303<br>EMD-51304<br>EMD-51305
**Data collection and processing**					
**Microscope**	Talos Arctica	Titan Krios			
**Magnification**	120,000	105,000			
**Voltage (kV)**	200	300			
**Electron exposure (e-/ Å^2)**	56.0	53.3			53.7
**Defocus range (µm)**	-1.2 to -2.4 µm				
**Pixel size (Å)**	1.21	0.34 (super-resolution) / 0.68 (native)			
**Symmetry imposed**	C1				
**No. micrographs**	4,573	5,540			5,656
**Initial particle images (no.)**	5,847,459	2,098,075			1,025,806
**Final particle images (no.)**	663,555	463,202	487,200	297,845	155,906
**Map resolution (Å)**	3.76	2.79	2.89	3.12	3.58
**FSC threshold**	0.143				
**Refinement**					
**Initial model used (PDB code)**	6R1T<br>6AZP (chain A)	6R1T	6R1T<br>1MHL	6R1T<br>1MHL	6R1T<br>1MHL
**Model resolution (Å)**	4.0	3.0		3.1	3.8
**FSC threshold**	0.5				
**Map sharpening *B* factor (Å^2)**	252.6	123.8	122.7	133.8	153.7
**Model composition**					
**Non-hydrogen atoms**	16,590	12,200	16,587	20,888	21,316
**Protein residues**	1,333	756	1326	1,897	1,889
**Nucleotides**	290	290	290	266	290
**Ligands**	2	-	3	22	20
**Water**	-	253	-	-	-
***B* factors (Å^2)**					
**Protein**	51.5	39.2	62.3	60.9	71.7
**Nucleotide**	69.7	35.0	46.4	64.0	111.4
**Ligand**	83.3	-	62.0	59.3	66.0
**Water**	-	31.2	-	-	-
**R.m.s. deviations**					
**Bond lengths (Å)**	0.003	0.005	0.003	0.003	0.003
**Bond angles (°)**	0.530	0.565	0.567	0.567	0.581
**Validation**					
**MolProbity score**	1.77	1.61	1.98	1.48	1.97
**Clashscore**	8.65	5.53	10.08	6.66	6.48
**Poor rotamers (%)**	1.94	2.55	3.10	1.41	2.95
**Ramachandran plot**					
**Favored (%)**	97.57	98.51	97.63	98.51	96.19
**Allowed (%)**	2.43	1.49	2.37	1.49	3.75
**Disallowed (%)**	0.00	0.00	0.00	0.00	0.05

Supplementary Table 2. Cryo-EM SPA datasets and models of reduced native MPO samples.

	Nucleosome bound by 1 MPO monomer	Nucleosome bound by 2 MPO monomers	Nucleosome bound by 1 MPO monomer and 1 MPO dimer
**PDB entry**	9IHD	9IHE	9IHF
**EMDB entry**	EMD-52865	EMD-52866	EMD-52867<br>EMD-52868<br>EMD-52869<br>EMD-52870
**Data collection and processing**			
**Microscope**	Talos Arctica		
**Magnification**	120,000		
**Voltage (kV)**	200		
**Electron exposure (e-/ Å^2)**	70.3		
**Defocus range (μm)**	-1.2 to -2.4		
**Pixel size (Å)**	0.68		
**Symmetry imposed**	C1		
**No. micrographs**	5,791		6,107
**Initial particle images (no.)**	2,666,393		2,909,302
**Final particle images (no.)**	160,726	185,760	53,312
**Map resolution (Å)**	2.97	2.95	3.16
**FSC threshold**	0.143		
**Refinement**			
**Initial model used (PDB code)**	9GEP	9GEP	9GEP
**Model resolution (Å)**	3.2	3.2	3.2
**FSC threshold**	0.5		
**Map sharpening *B* factor (Å^2)**	102.9	86.8	69.1
**Model composition**			
**Non-hydrogen atoms**	16,508	21,069	25,213
**Protein residues**	1,313	1,870	2,424
**Nucleotides**	290	290	266
**Ligands**	4	8	21
**Water**	-	-	-
***B* factors (Å^2)**			
**Protein**	59.5	77.4	77.4
**Nucleotide**	29.3	45.6	76.2
**Ligand**	71.0	88.2	87.7
**Water**	-	-	-
**R.m.s. deviations**			
**Bond lengths (Å)**	0.004	0.004	0.003
**Bond angles (°)**	0.583	0.556	0.518
**Validation**			
**MolProbity score**	1.70	1.54	1.56
**Clashscore**	6.76	7.13	6.98
**Poor rotamers (%)**	2.69	1.49	1.53
**Ramachandran plot**			
**Favored (%)**	98.1	97.9	97.8
**Allowed (%)**	1.9	2.1	2.2
**Disallowed (%)**	0.0	0.0	0.0

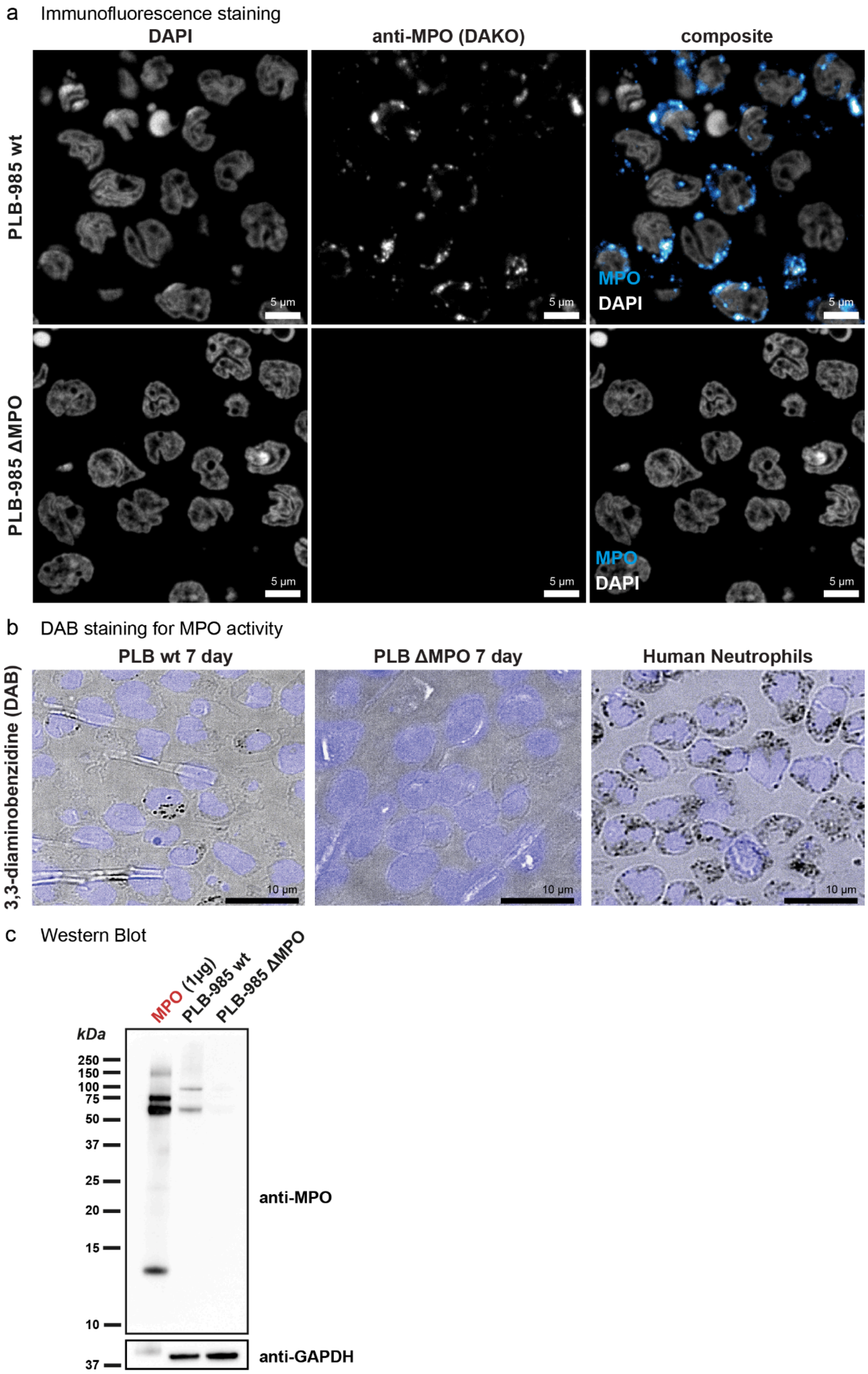
a
Immunofluorescence staining
DAPI
anti-MPO (DAKO)
composite
PLB-985 wt
PLB-985 ΔMPO
MPO
DAPI
5 µm
b
DAB staining for MPO activity
PLB wt 7 day
PLB ΔMPO 7 day
Human Neutrophils
3,3-diaminobenzidine (DAB)
10 µm
c
Western Blot
MPO (1µg)
PLB-985 wt
PLB-985 ΔMPO
kDa
250
150
100
75
50
37
25
20
15
10
37
anti-MPO
anti-GAPDH

Supplementary Figure 1. Antibody verification using MPO knockout cell line. a, Immunofluorescent staining of wildtype and MPO knockout PLB-985 labeled with MPO (DAKO A0398) pAb. Scale bar = 5μm. **b,** Fixed neutrophils were treated with 3,3′-Diaminobenzidine (DAB) which precipitates in the presence of MPO activity. Precipitates were then visualised using light microscopy. **c,** Lysates of wildtype and MPO knockout PLB-985 and purified human native MPO were immuno-blotted with anti-MPO (DAKO A0398). The uncropped blots are shown in **Supplementary Fig. 29**.

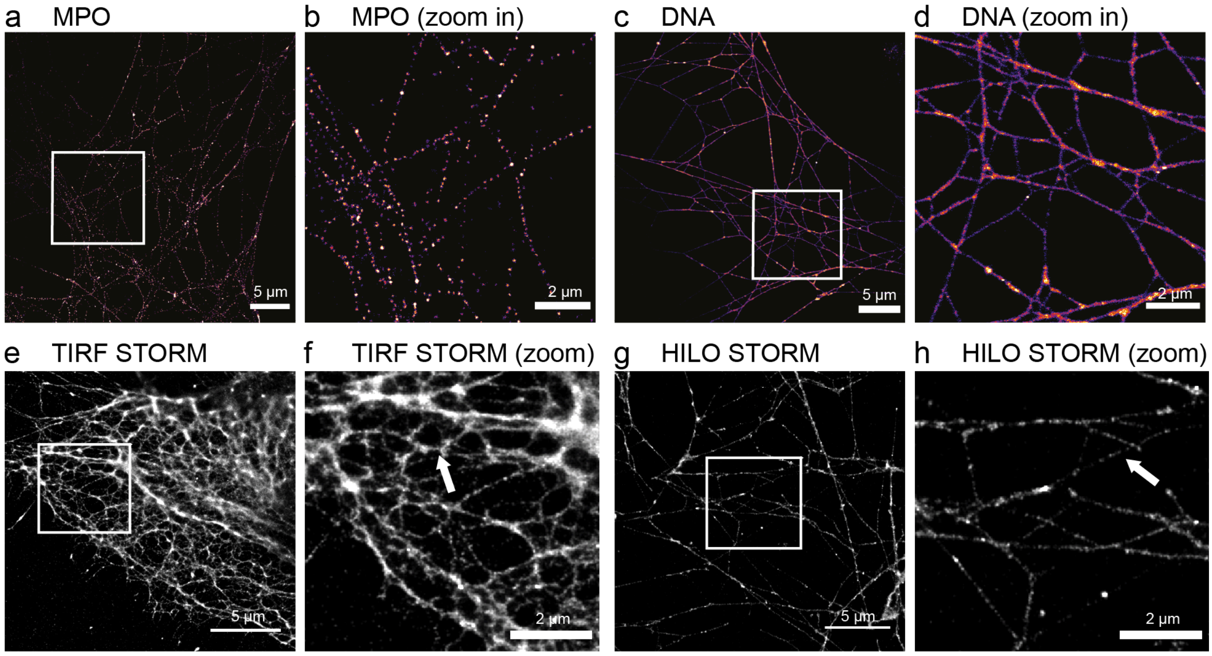

Supplementary Figure 2. Single color STORM images using anti-MPO or anti-DNA antibodies and acquisition using HILO. a, STORM image of MPO on NETs discontinuous staining patterns to be directly compared to continuous staining of filaments with an anti-DNA antibody in panels **c** and **d**. **b,** Zoomed in view of panel **a**. **c,** STORM image of continuous DNA staining on NET filaments. **d,** Zoomed in view of panel **c**. **e,** TIRF-STORM image of NET filaments stained with an antibody PL2.3 against an H2A/H2B/DNA complex. **f,** Zoomed in view of panel **e**. **g,** HILO-STORM image of NET filaments stained with an antibody PL2.3 against an H2A/H2B/DNA complex. **h,** Zoomed in view of panel **g**.

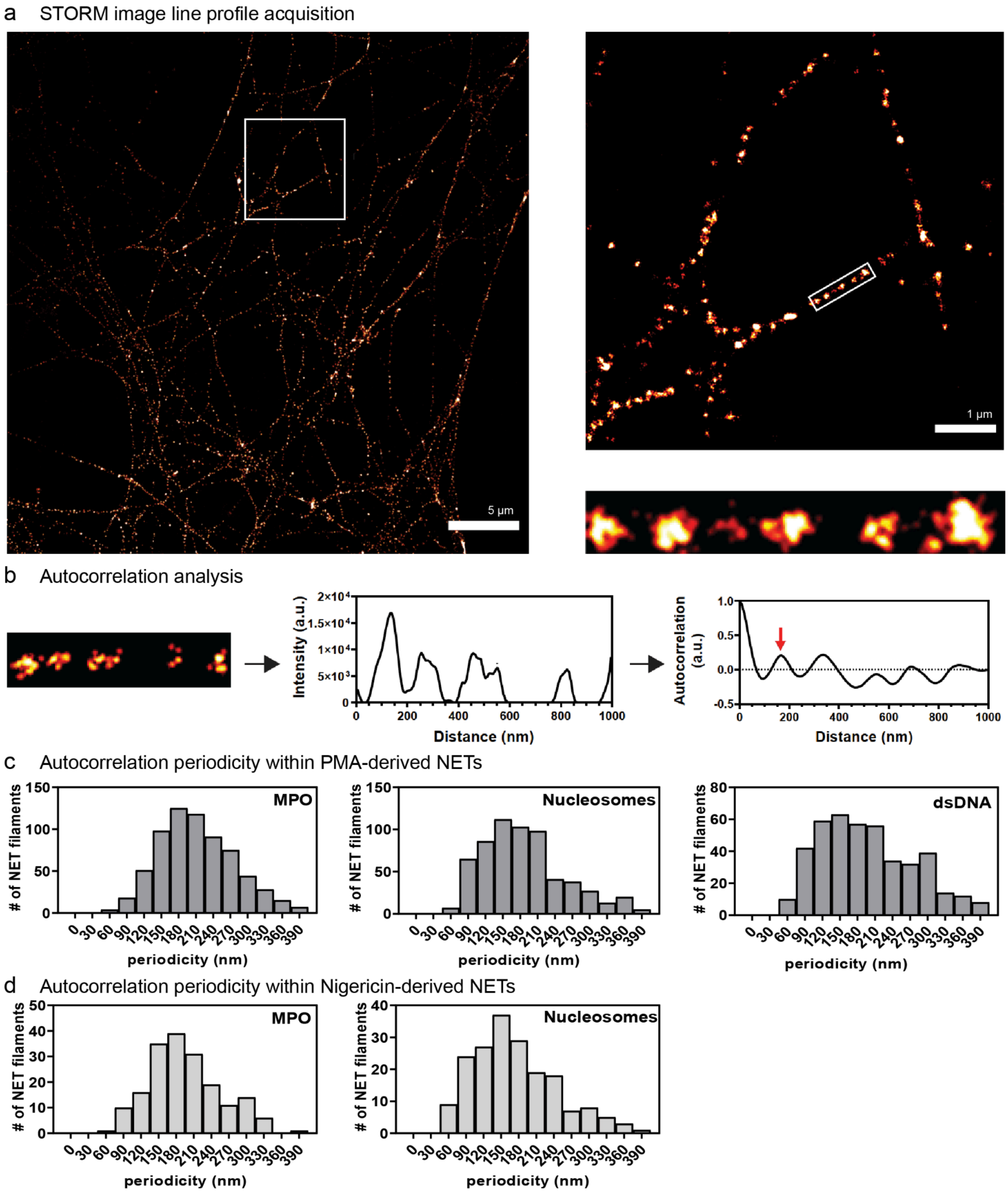

Supplementary Figure 3. Autocorrelation analysis of PMA or Nigericin induced NETs. a, STORM image line profile acquisition. Scale bar 5μm (left panel) and 1μm (right panel). **b,** Line profile visualization where x=distance and y=intensity (left) and autocorrelation analysis based on auto-templating of line profile where x=distance and y=autocorrelation with 1 being perfectly autocorrelated and -1 being anticorrelated. The first peak was used to generate autocorrelation values. **c,** Autocorrelation of MPO/Nucleosome (from **Fig. 1b,d** using different bins did not change results) and double stranded DNA from PMA stimulated NETS. *n*=6

independent experiments (71992 NET filament fragments analyzed). **d,** NET stimulus Nigericin demonstrated similar periodicity values as compared to PMA for MPO (192 NET filament fragments analyzed) and nucleosome (160 NET filament fragments analyzed). *n*=1 experiment.

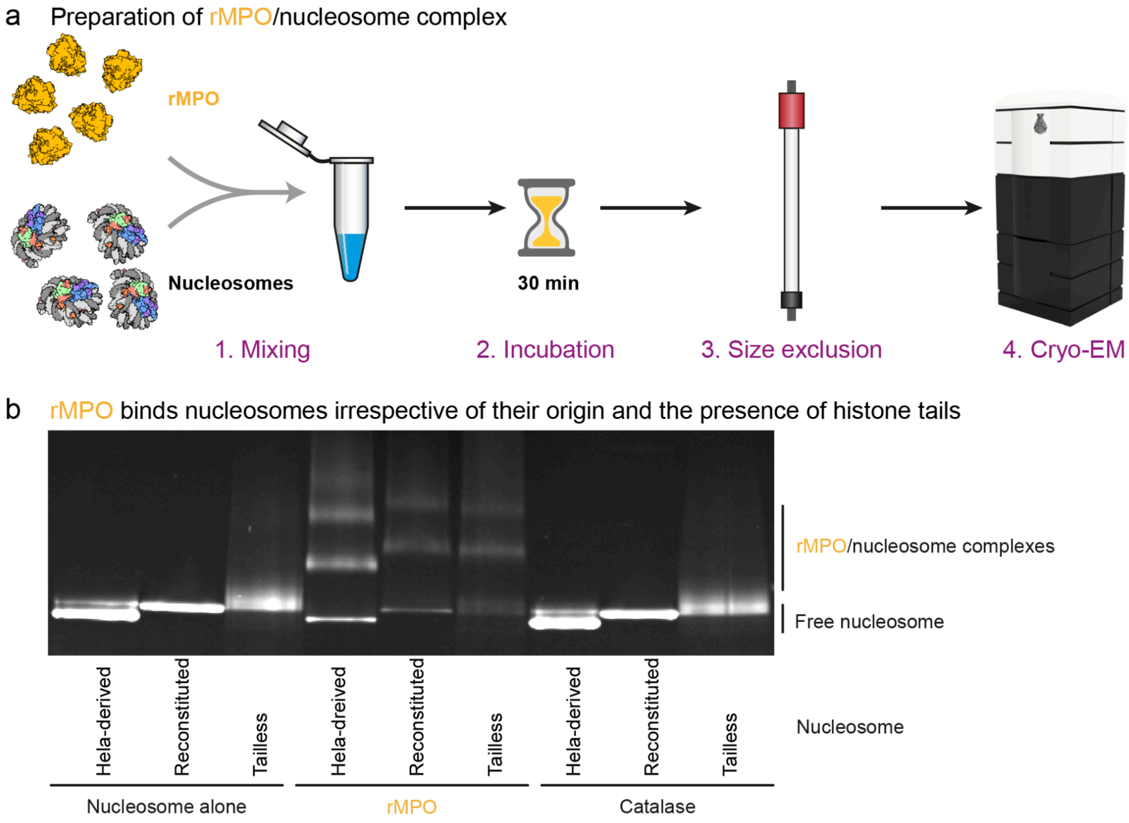

Supplementary Figure 4. rMPO forms stable and purifiable complexes with nucleosomes. a, Scheme showing the reconstitution of complexes comprising rMPO and nucleosomes by mixing (1) and incubation (2), followed by purification using size exclusion chromatography via a Superdex 200 5/150 column (3) and subsequent analysis by cryo-EM (4). **b,** Native gel shift assay. Mononucleosomes either derived from HeLa cells or recombinantly reconstituted either using full-length or truncated, tailless histones, and recombinant MPO or catalase were subjected to native gel shift assay after 10 minutes of incubation at room temperature to monitor MPO-nucleosome interactions. *n*=2 independent experiments. The uncropped gel is shown in **Supplementary Fig. 29**.

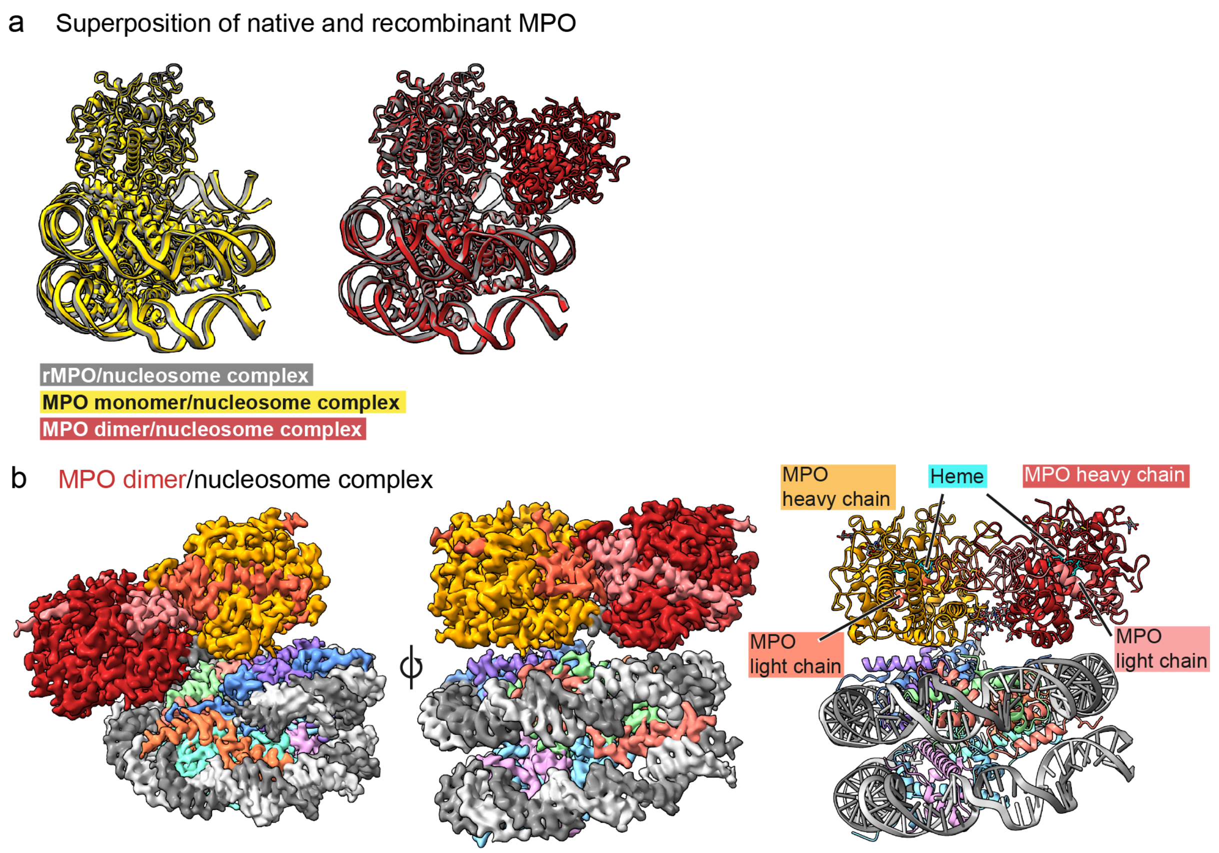

Supplementary Figure 5. Similar nucleosome binding mode of MPO via acidic patch in different complex arrangements. a, Superposition of the rMPO/nucleosome complex (grey) with MPO/nucleosome complexes containing MPO monomers (yellow) and dimers (red), respectively. RMSDs: 1.26 Å over 1584 residues for dimer/nucleosome vs. monomer/nucleosome; 0.74 Å over 1584 residues for dimer/nucleosome vs. rMPO/nucleosome. **b,** Cryo-EM reconstruction (in two orientations) and model of MPO dimer/nucleosome complex.

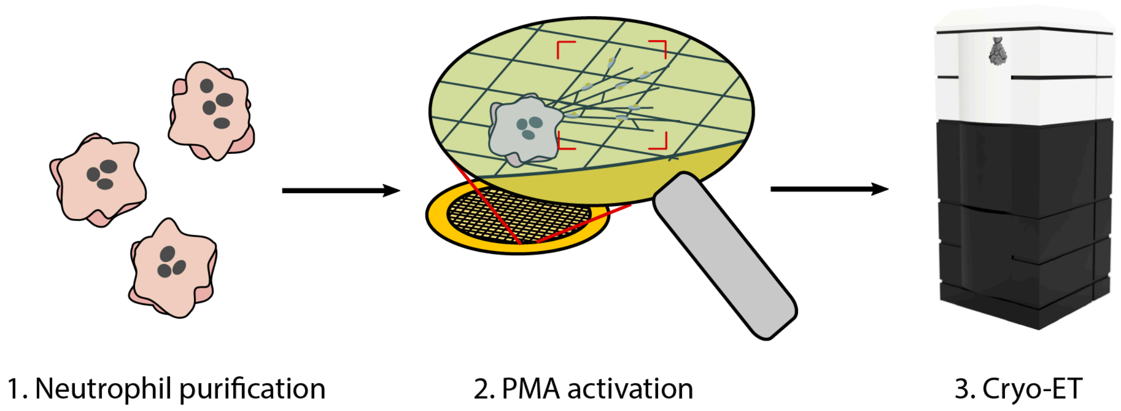

Supplementary Figure 6. Sample preparation for *in-situ* structure determination by cryo-ET. Scheme of the sample preparation for the cryo-ET experiments. After neutrophil purification, cells were activated with PMA on cryo-EM grids. After vitrification, grids were screened and finally, tomograms were taken in the periphery of neutrophils which showed clear NETosis.

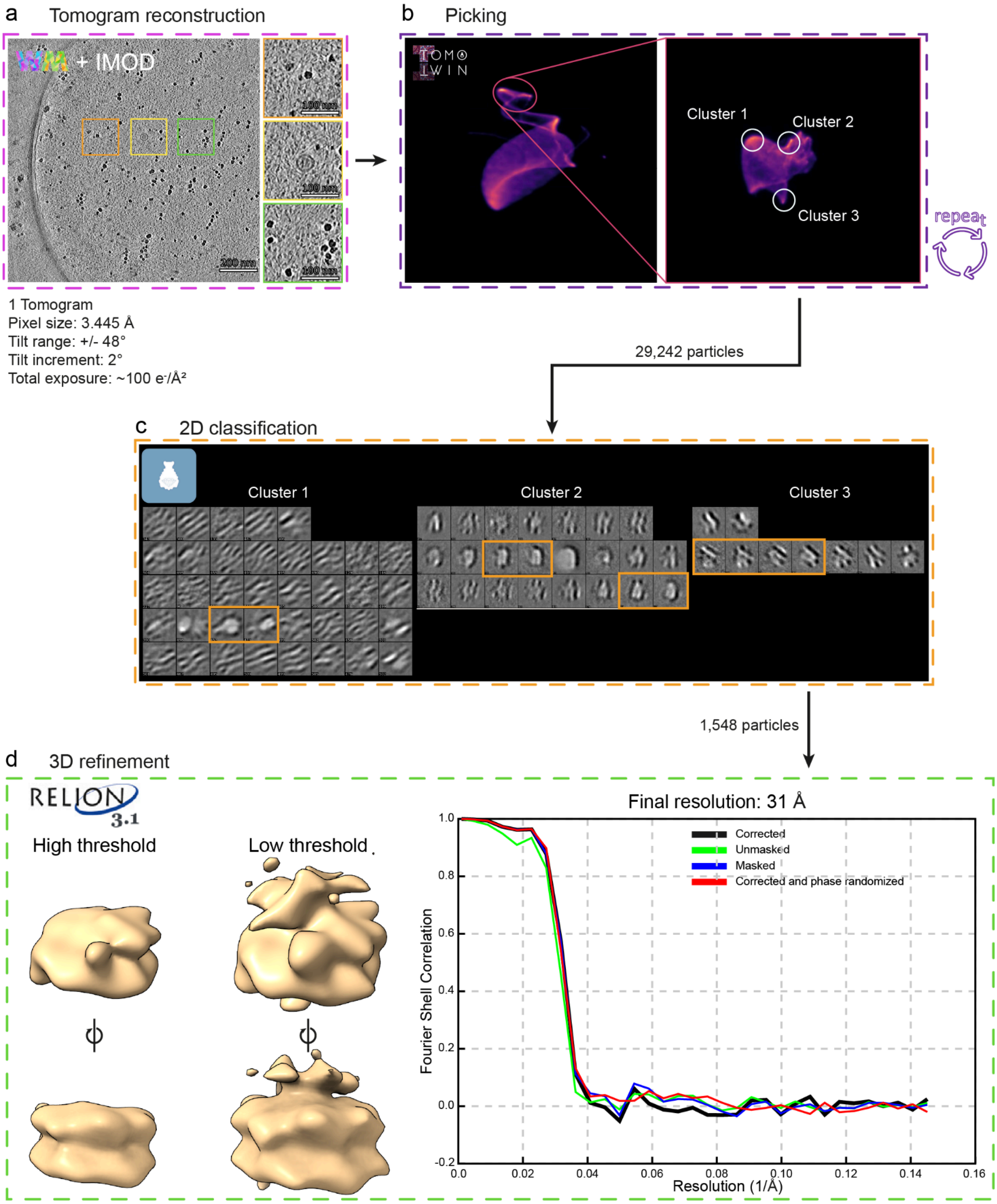

Supplementary Figure 7. Cryo-ET data processing. a, Tomograms were pre-processed in Warp reconstructed using IMOD. **b,** Particles were picked and clustered using TomoTwin, followed by several rounds of iterative UMAP polishing. **c,** Extracted particles of the three identified clusters were separately subjected to 2D classification in SPHIRE. **d,** Particles of cluster 3 associated with good 2D classes were pooled. The subsequent 3D refinement in Relion yielded a resolution of 31Å.

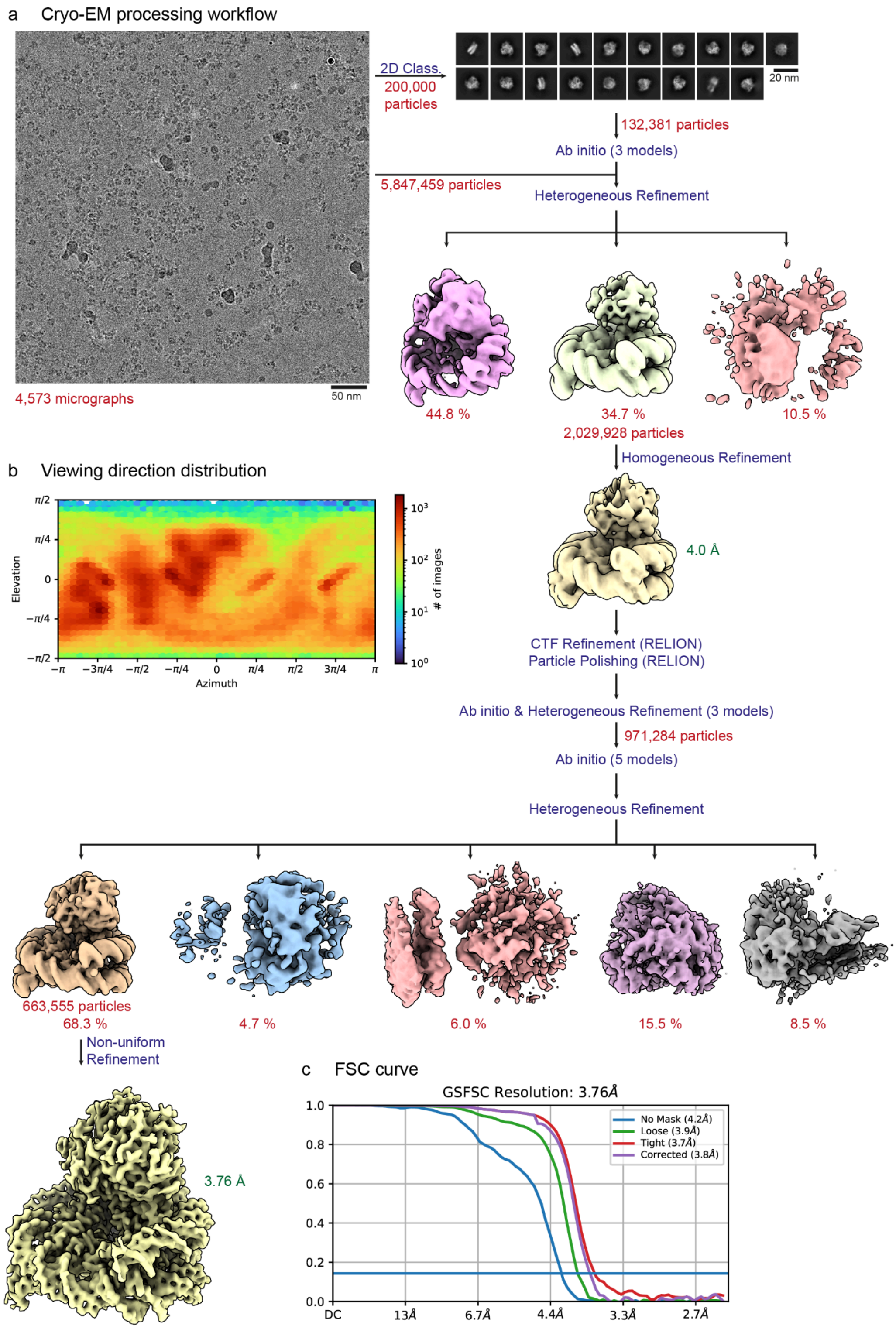

Supplementary Figure 8. Cryo-EM data processing of rMPO/nucleosome dataset. a, Processing workflow including a representative micrograph, 2D classes and intermediate and final reconstructions. The final reconstruction reached 3.76 Å. **b,** Particle orientation distribution plot. **c,** Gold-standard FSC curve. The blue horizontal line marks the gold-standard 0.143 threshold.

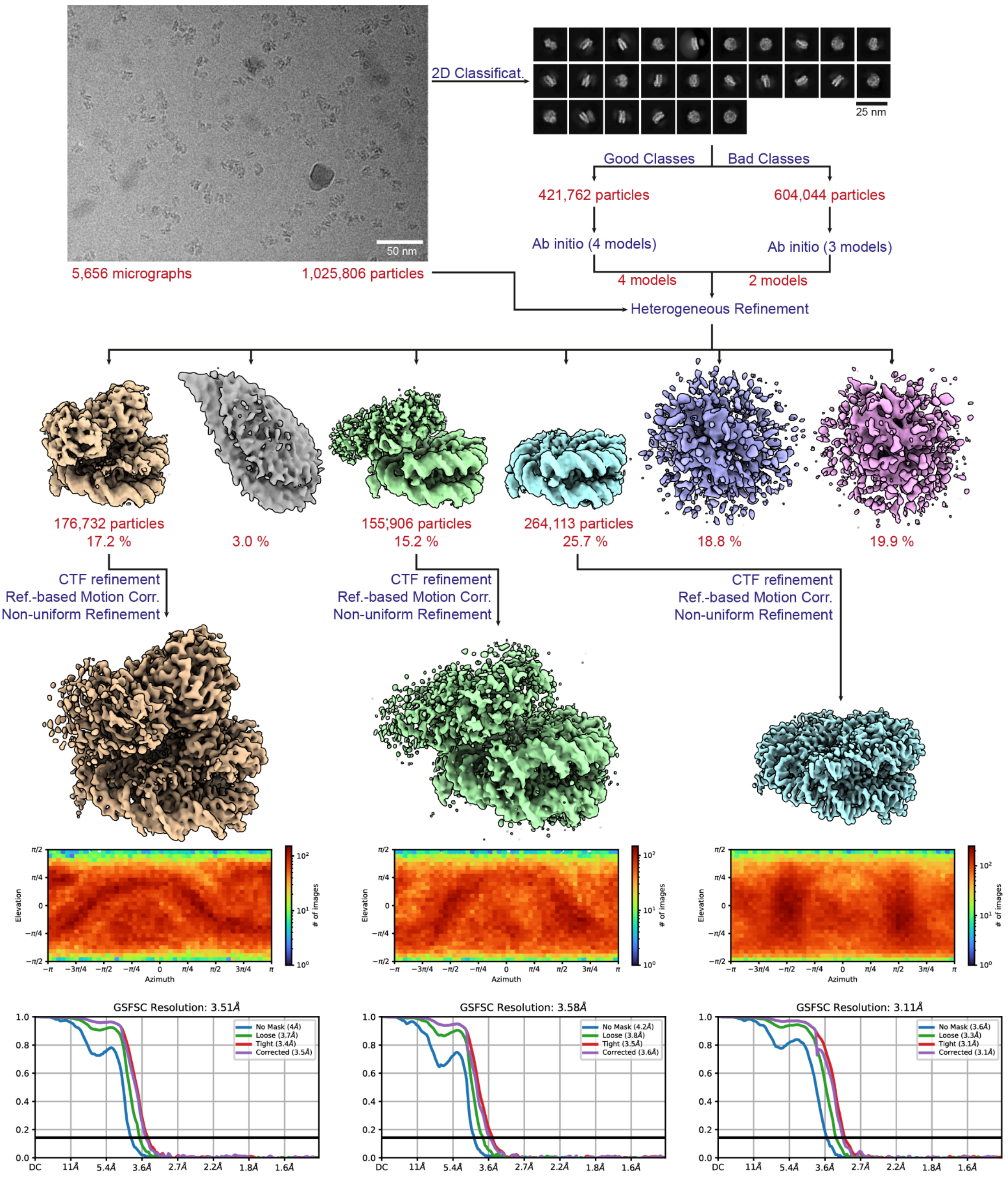

Supplementary Figure 9. Cryo-EM data processing of 15 seconds time point of the MPO/nucleosome complex. The processing workflow includes a representative micrograph, 2D classes and intermediate and final reconstructions. The final reconstructions reached 3.51 Å (MPO dimer/nucleosome), 3.58 Å (MPO dimer/nucleosome, intermediate arrangement) and 3.11 Å (free nucleosome), respectively.

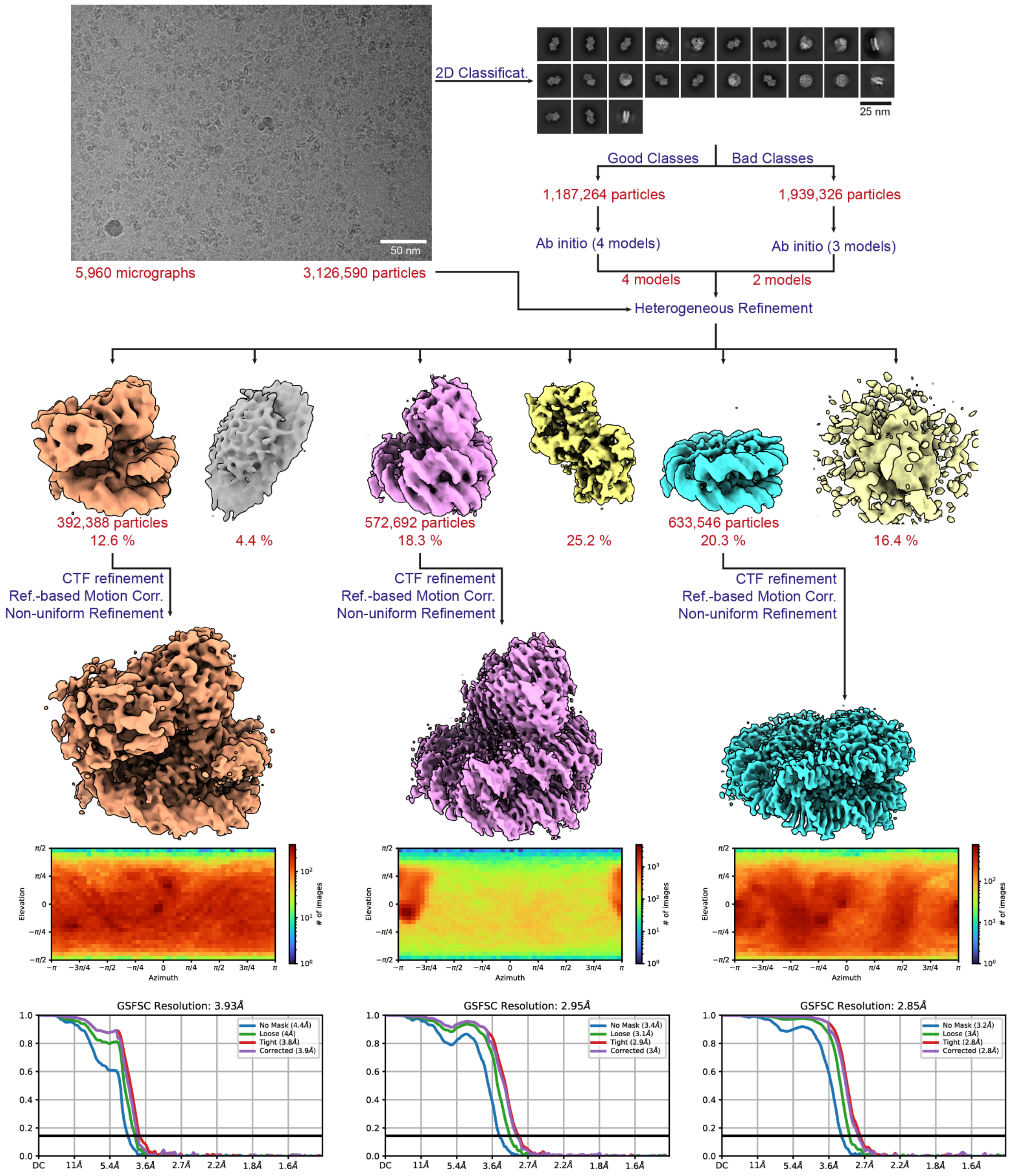

Supplementary Figure 10. Cryo-EM data processing of 2 minutes time point of the MPO/nucleosome complex. The processing workflow includes a representative micrograph, 2D classes and intermediate and final reconstructions. The final reconstructions reached 3.93 Å (MPO dimer/nucleosome), 2.95 Å (MPO monomer/nucleosome) and 2.85 Å (free nucleosome), respectively.

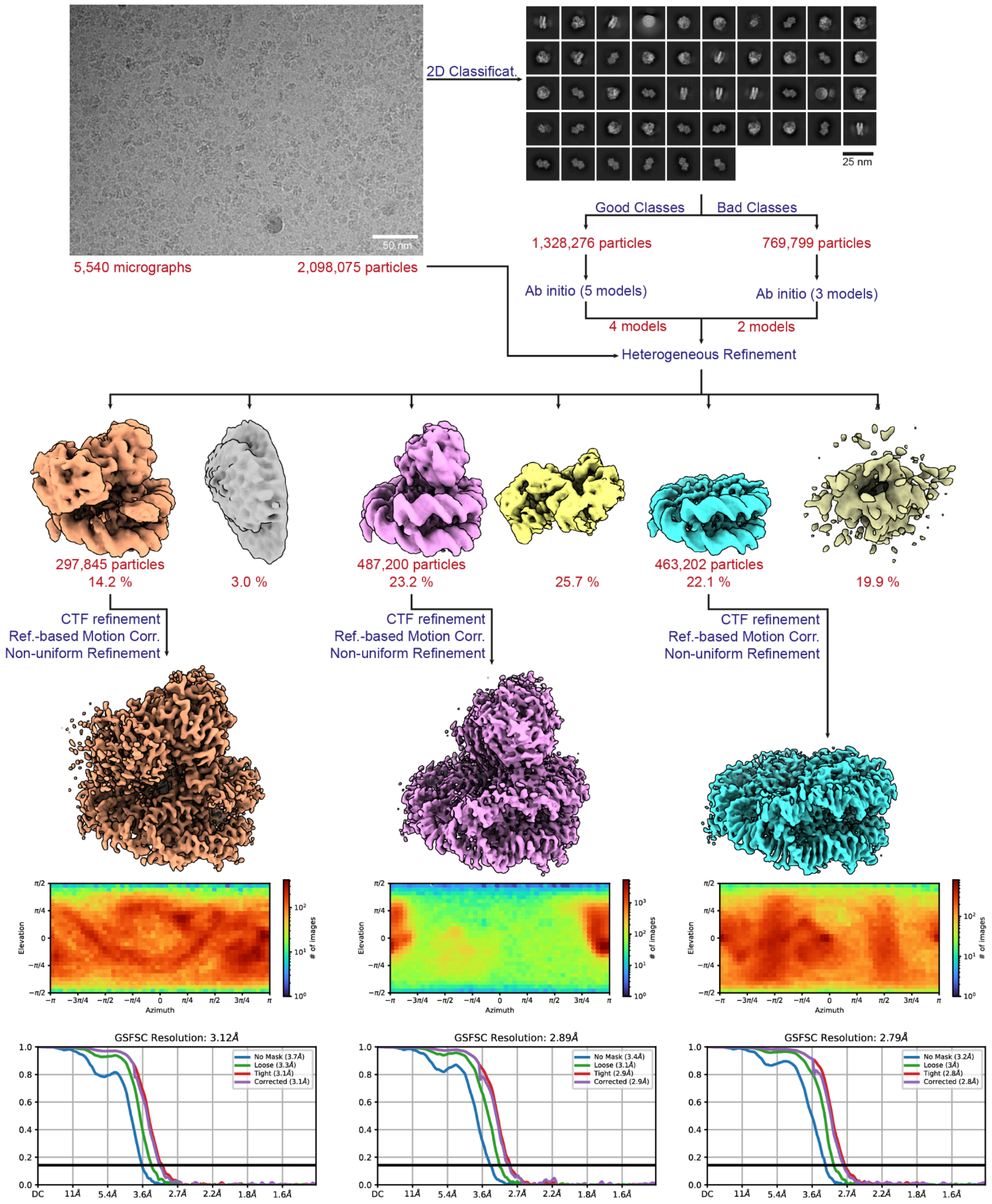

Supplementary Figure 11. Cryo-EM data processing of 5 minutes time point of the MPO/nucleosome complex. The processing workflow includes a representative micrograph, 2D classes and intermediate and final reconstructions. The final reconstructions reached 3.12 Å (MPO dimer/nucleosome), 2.89 Å (MPO monomer/nucleosome) and 2.79 Å (free nucleosome), respectively.

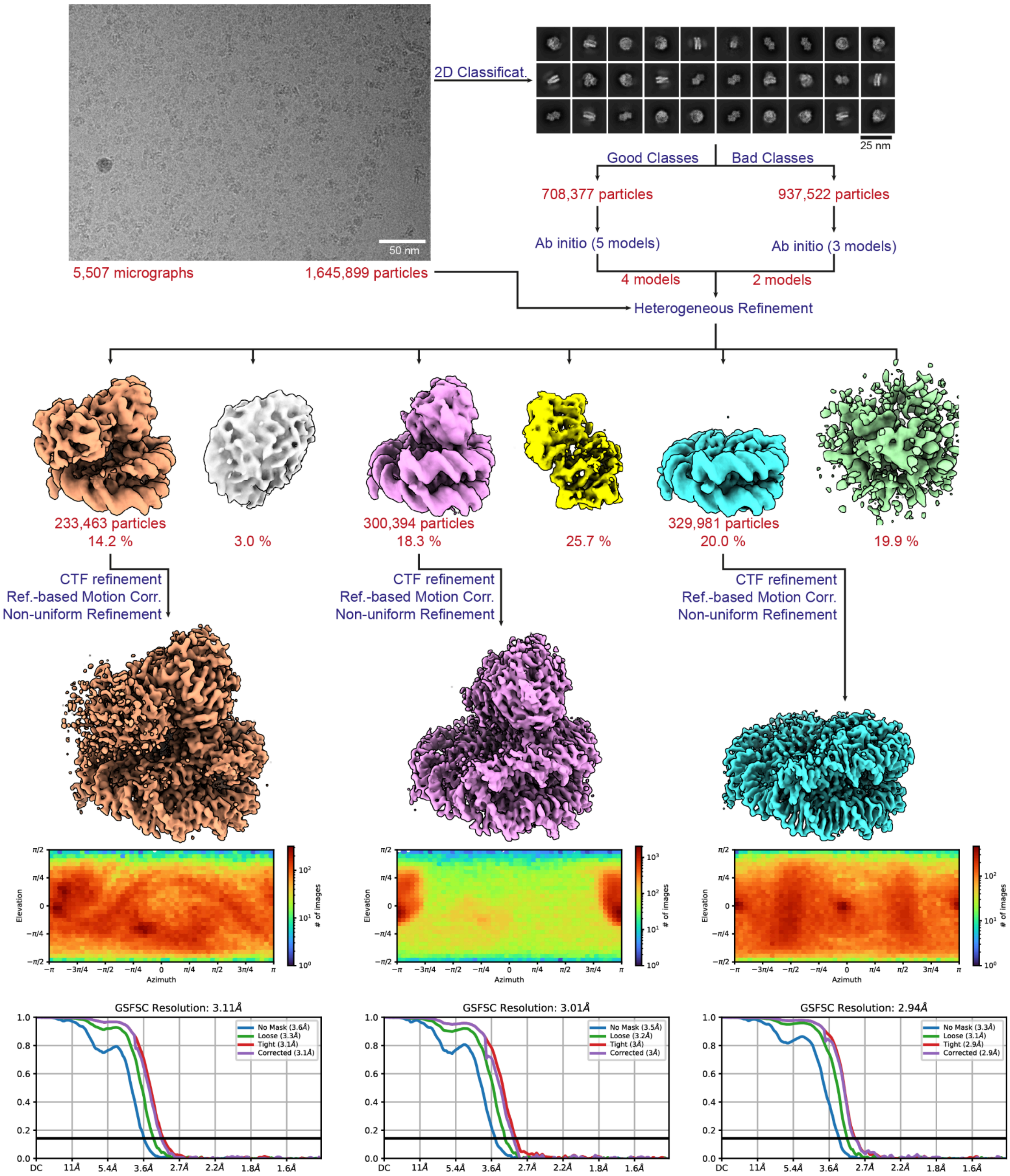

Supplementary Figure 12. Cryo-EM data processing of 10 minutes time point of the MPO/nucleosome complex. The processing workflow includes a representative micrograph, 2D classes and intermediate and final reconstructions. The final reconstructions reached 3.11 Å (MPO dimer/nucleosome), 3.01 Å (MPO monomer/nucleosome) and 2.94 Å (free nucleosome), respectively.

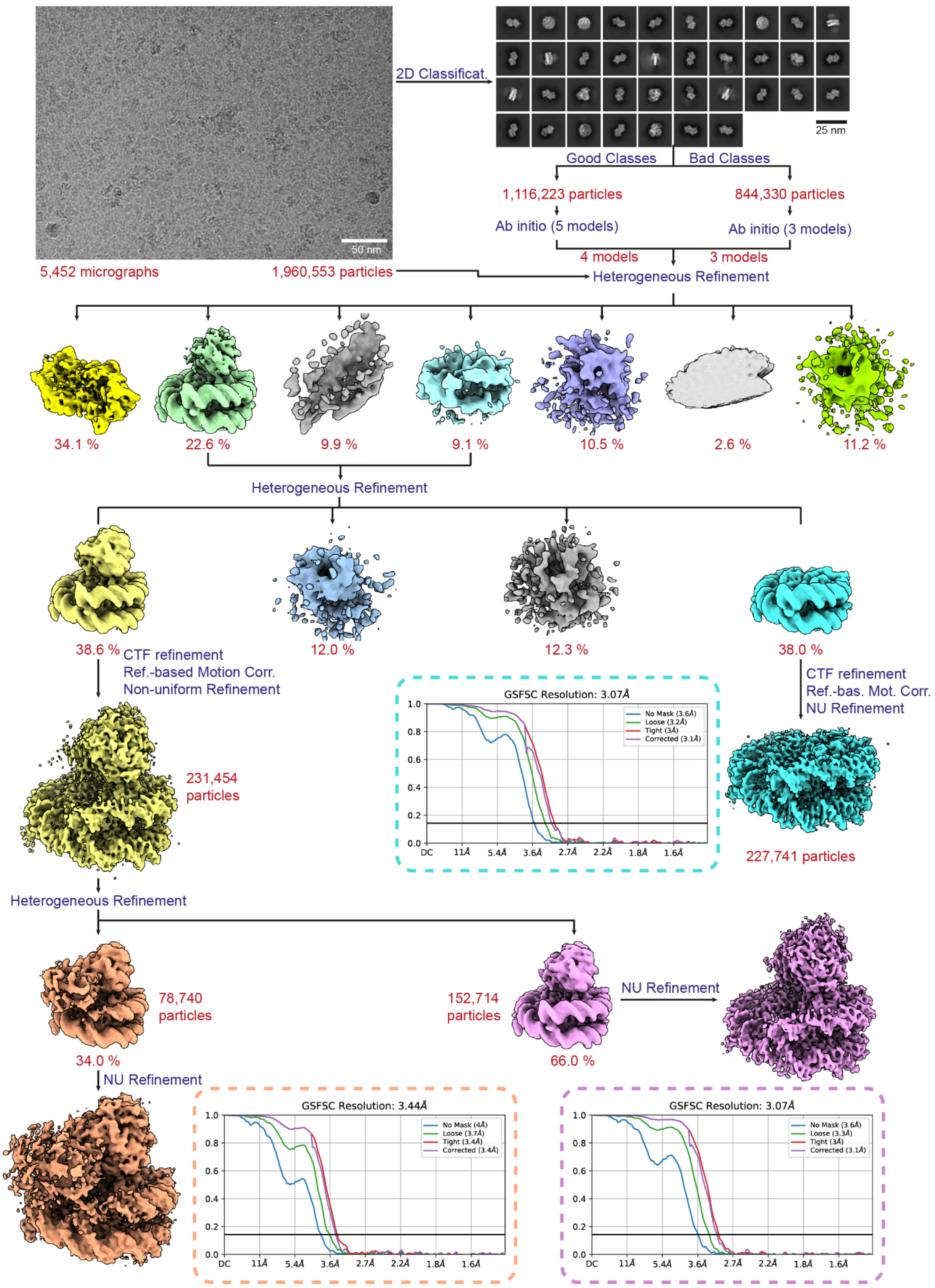

Supplementary Figure 13. Cryo-EM data processing of 20 minutes time point of the MPO/nucleosome complex. The processing workflow includes a representative micrograph, 2D classes and intermediate and final reconstructions. The final reconstructions reached 3.44 Å (MPO dimer/nucleosome), 3.07 Å (MPO monomer/nucleosome) and 3.07 Å (free nucleosome), respectively.

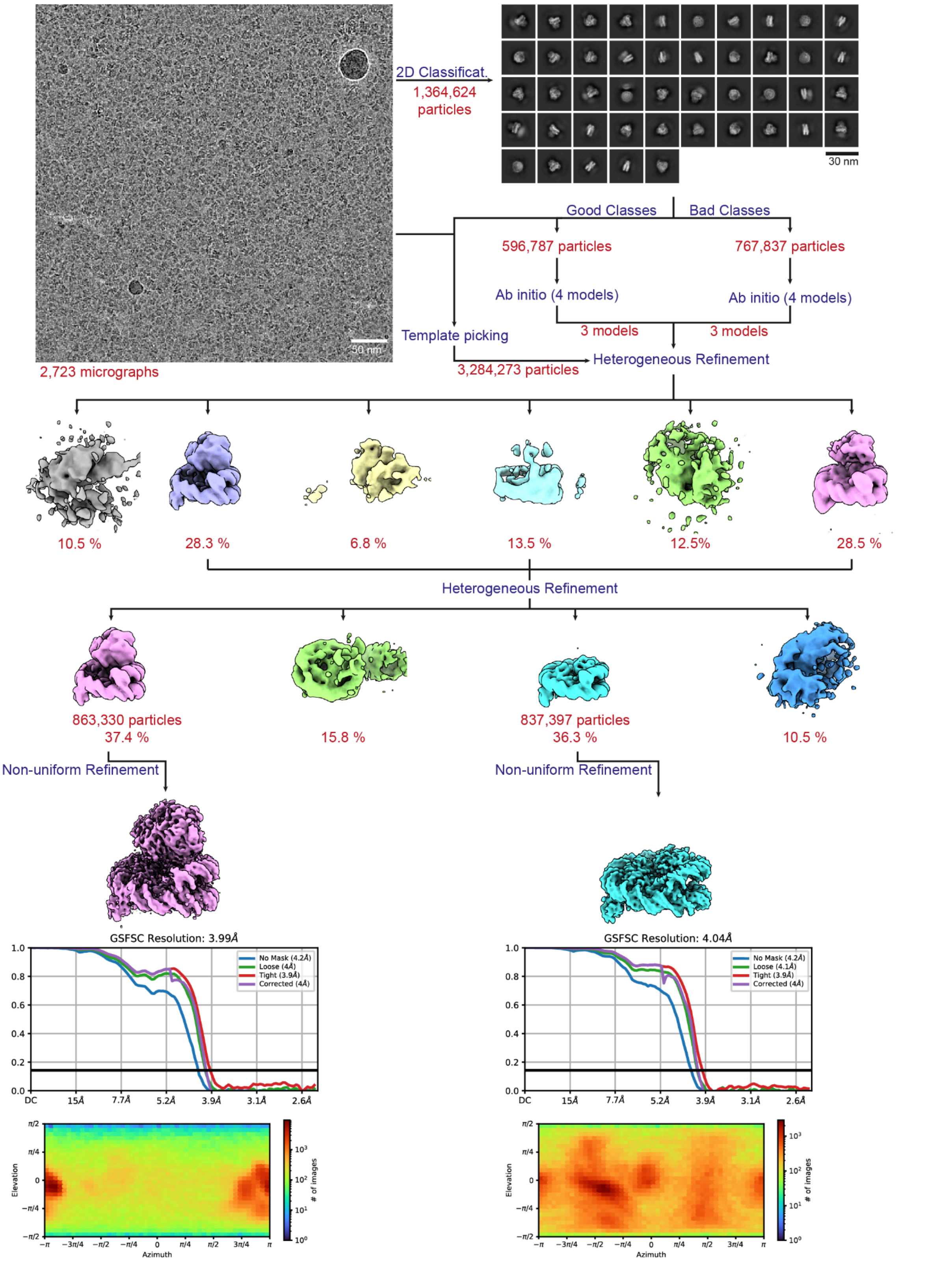

Supplementary Figure 14. Cryo-EM data processing of the nMPO/nucleosome complex after 30 minute incubation and size exclusion chromatography. The processing workflow includes a representative micrograph, 2D classes and intermediate and final reconstructions. The final reconstructions reached 3.99 Å (MPO monomer/nucleosome complex) and 4.04 Å (free nucleosome), respectively.

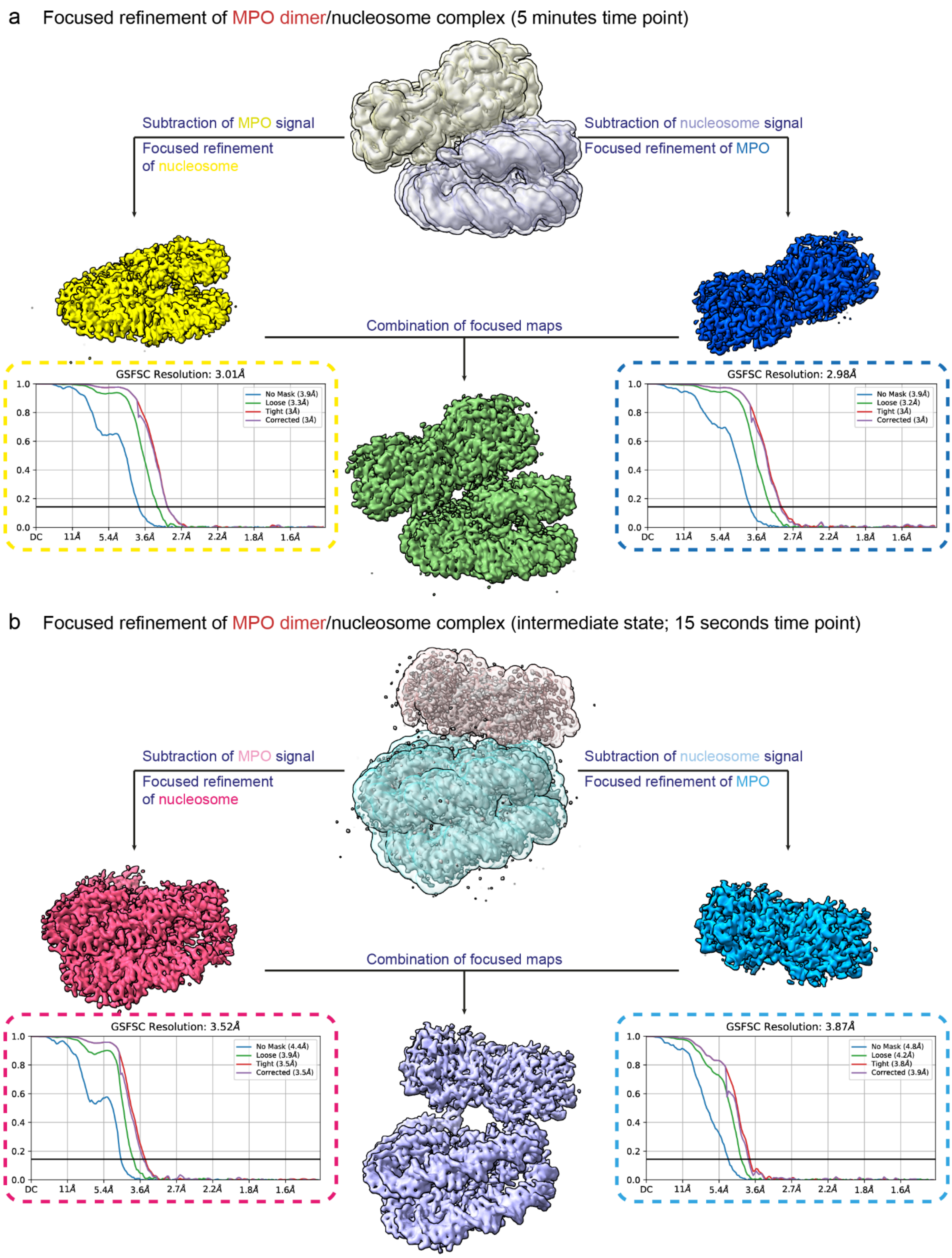

Supplementary Figure 15. Focused refinement procedures. a, Focused refinement of the MPO dimer/nucleosome complex (time course; 5 minutes time point). The globally refined particles were subjected to signal subtraction removing either the MPO signal or the nucleosome signal. Then, they were refined using a reference corresponding to the remaining part of the structure. These refinements

resulted in improved 3.01 Å (nucleosome) and 2.98 Å (MPO) reconstructions, as opposed to the 3.12 Å of the global refinement. Finally, both focused maps were combined in ChimeraX. **a,** Focused refinement of the intermediate conformation of the MPO dimer/nucleosome complex that was only found in the 15 seconds dataset. The strategy was similar to what is described in panel **a**.

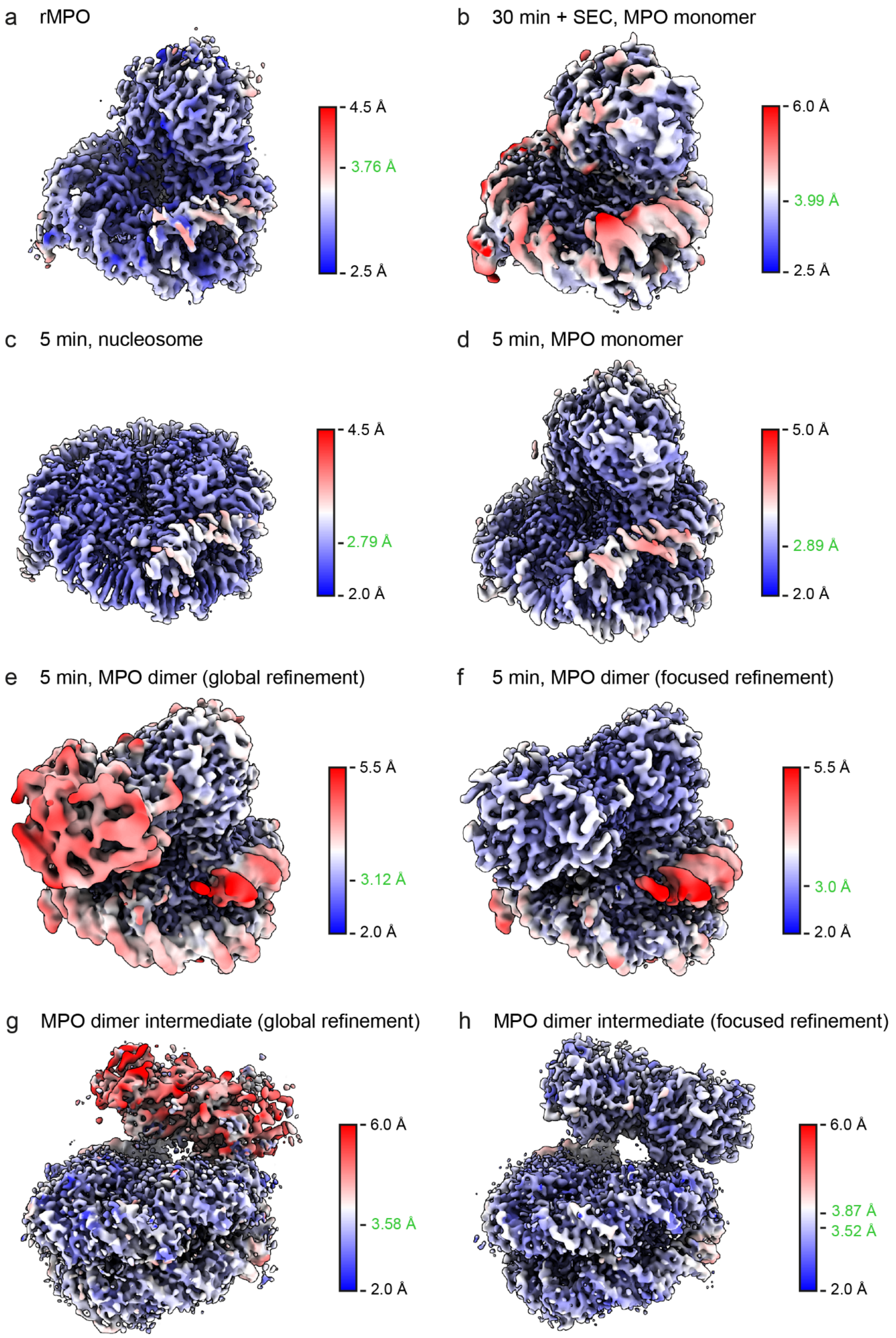

Supplementary Fig. 16. Local resolution of cryo-EM reconstructions. Local resolution was calculated and maps locally filtered using cryoSPARC. Local resolution is shown as a color gradient from red (low resolution) over white to blue (high resolution) on the isosurface of the reconstructed cryo-EM maps. Comparison of panels **e** with **f** and **g** with **h**, respectively, show the improvement of local resolution upon focused refinement.

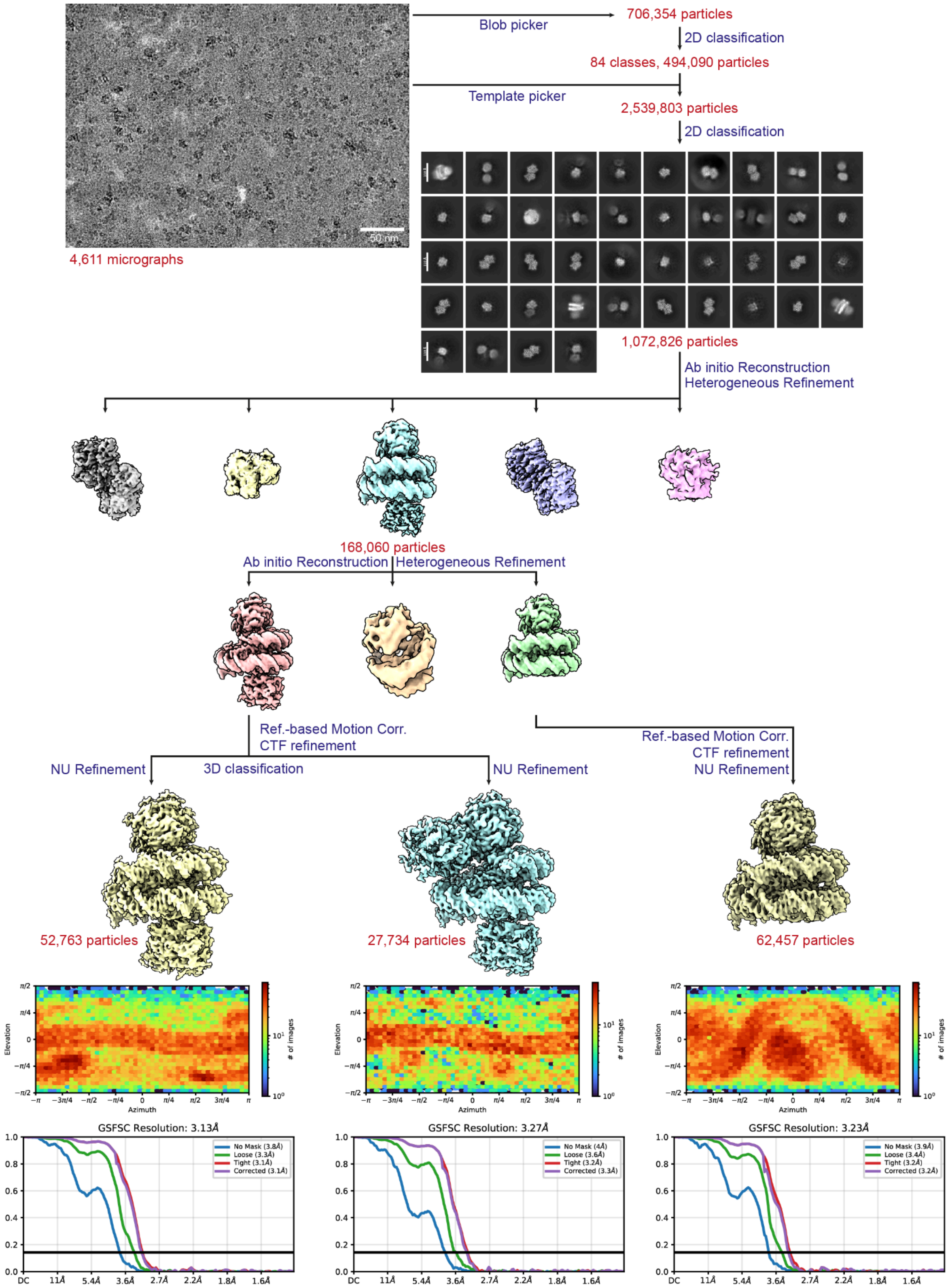

Supplementary Figure 17. Cryo-EM data processing of 15 seconds time point of the reduced MPO/nucleosome complex. The processing workflow includes a representative micrograph, 2D classes and intermediate and final reconstructions. The final reconstructions reached 3.13 Å (nucleosome bound by two MPO monomers), 3.27 Å (nucleosome bound by one MPO monomer and one MPO dimer) and 3.23 Å (nucleosome bound by one MPO monomer), respectively.

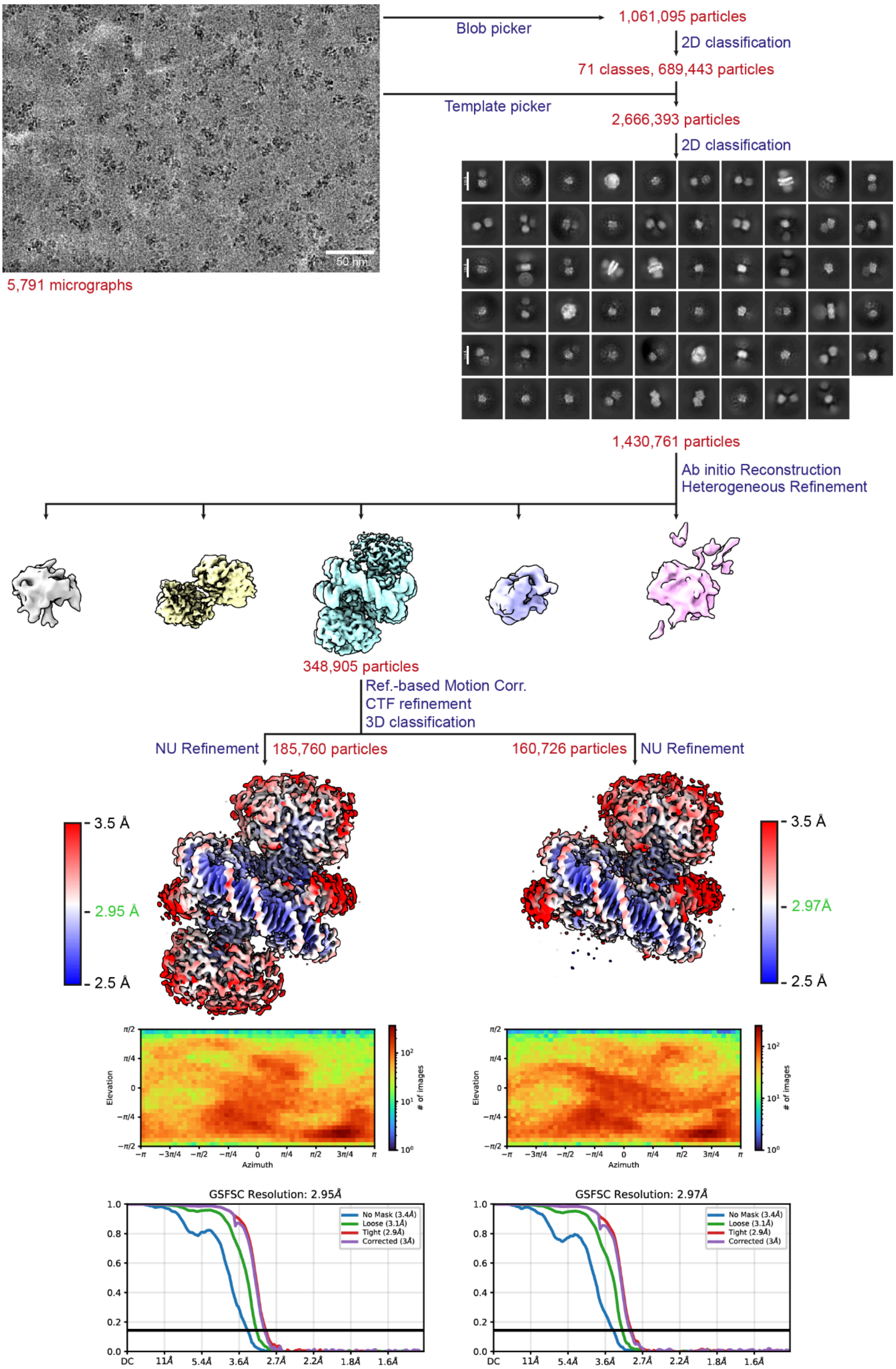

Supplementary Figure 18. Cryo-EM data processing of 2 minutes time point of

the reduced MPO/nucleosome complex. The processing workflow includes a representative micrograph, 2D classes and intermediate and final reconstructions. The final reconstructions reached 2.95 Å (nucleosome bound by two MPO monomers) and 2.97 Å (nucleosome bound by one MPO monomer), respectively.

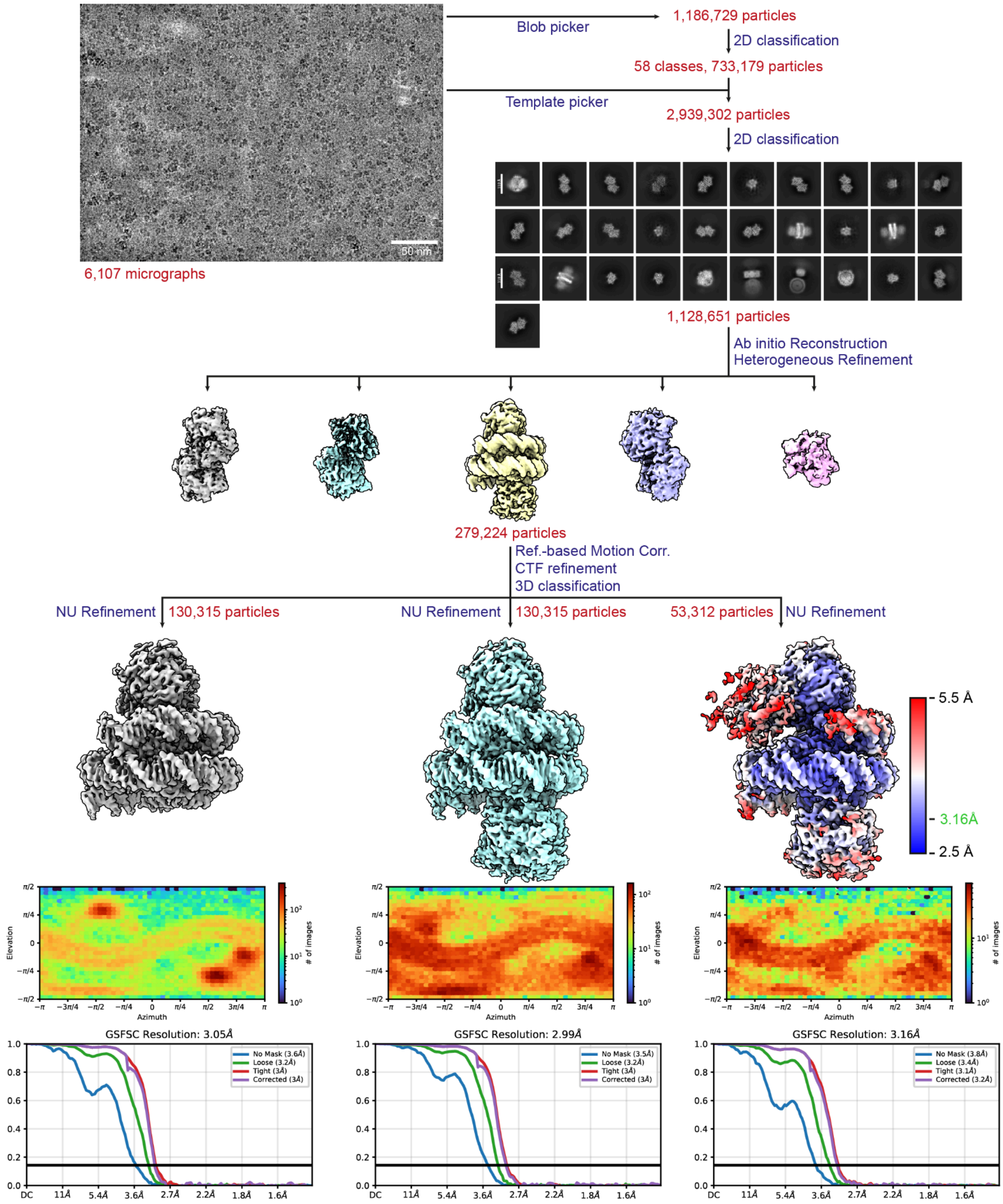

Supplementary Figure 19. Cryo-EM data processing of 5 minutes time point of the reduced MPO/nucleosome complex. The processing workflow includes a representative micrograph, 2D classes and intermediate and final reconstructions. The final reconstructions reached 3.05 Å (nucleosome bound by one MPO monomer), 2.99 Å (nucleosome bound by two MPO monomers) and 3.16 Å (nucleosome bound by one MPO monomer and one MPO dimer), respectively.

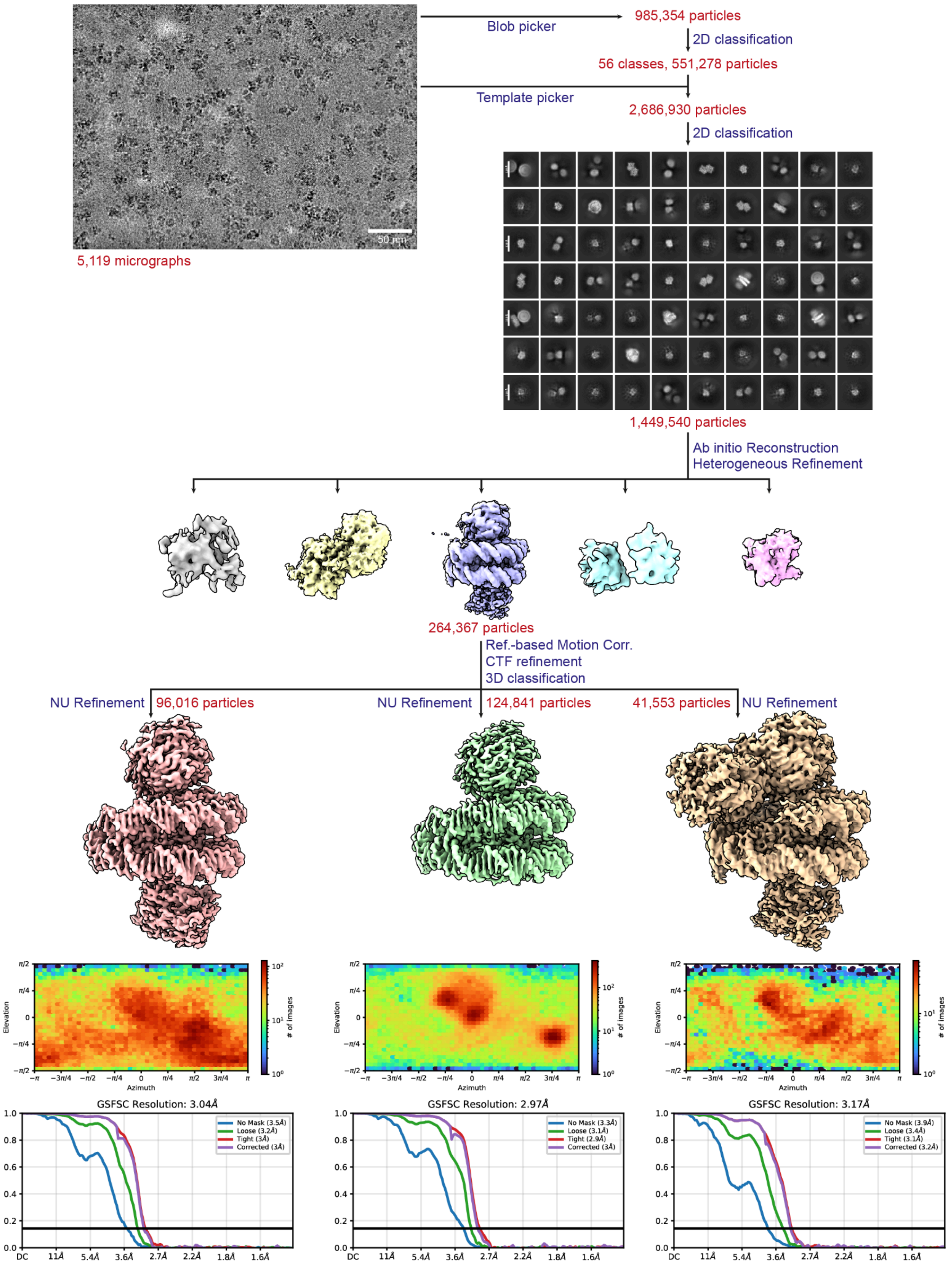

Supplementary Figure 20. Cryo-EM data processing of 60 minutes time point of the reduced MPO/nucleosome complex. The processing workflow includes a representative micrograph, 2D classes and intermediate and final reconstructions. The final reconstructions reached 3.04 Å (nucleosome bound by two MPO monomers), 2.97 Å (nucleosome bound by one MPO monomer) and 3.17 Å (nucleosome bound by one MPO monomer and one MPO dimer), respectively.

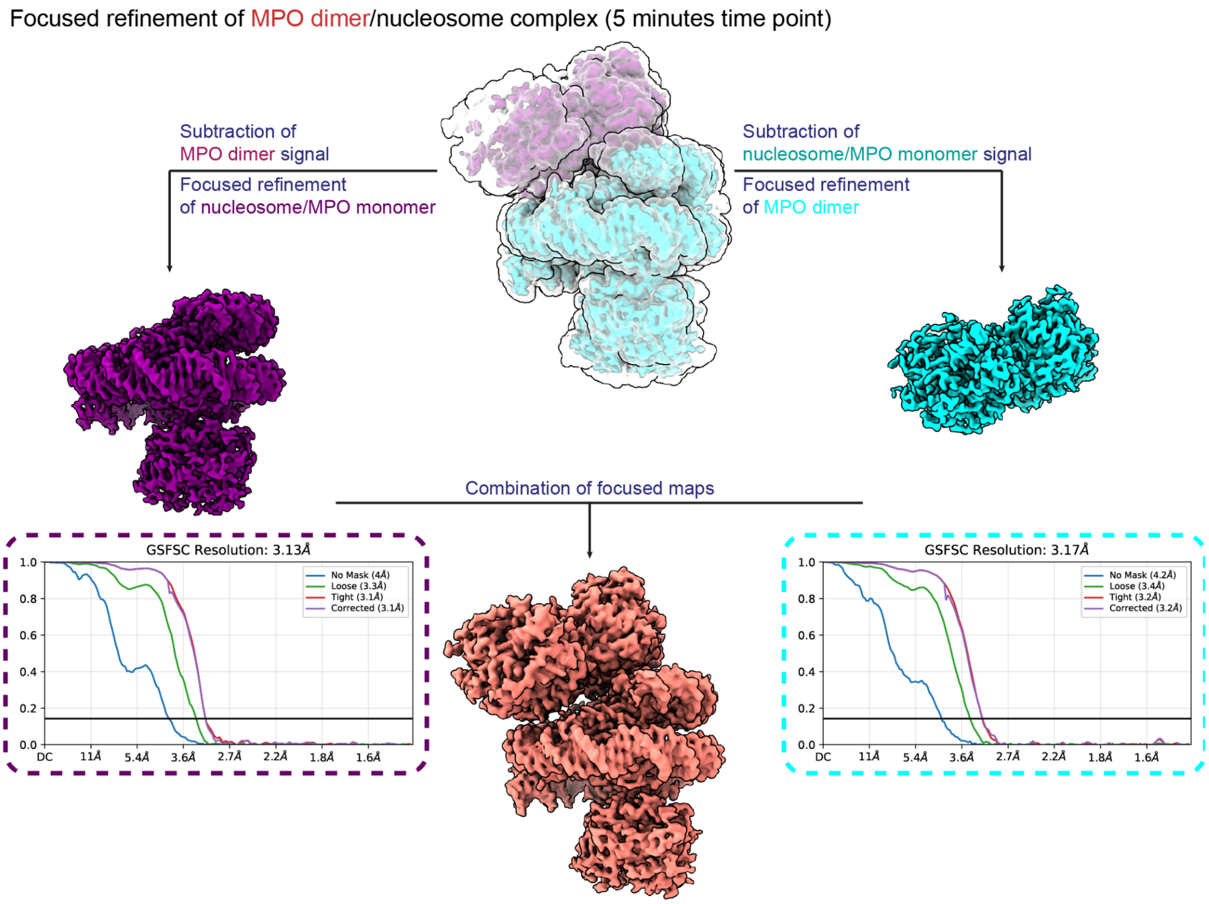

Supplementary Figure 21. Focused refinement procedures. Focused refinement of the Nucleosome bound by one MPO monomer and one MPO dimer (time course; 5 minutes time point). The globally refined particles were subjected to signal subtraction removing the signal of either the MPO dimer or the MPO monomer/nucleosome subcomplex. Then, they were refined using a reference corresponding to the remaining part of the structure. These refinements resulted in improved 3.13 Å (MPO monomer/nucleosome) and 3.17 Å (MPO dimer) reconstructions, as opposed to the 3.16 Å of the global refinement. Finally, both focused maps were combined in ChimeraX.

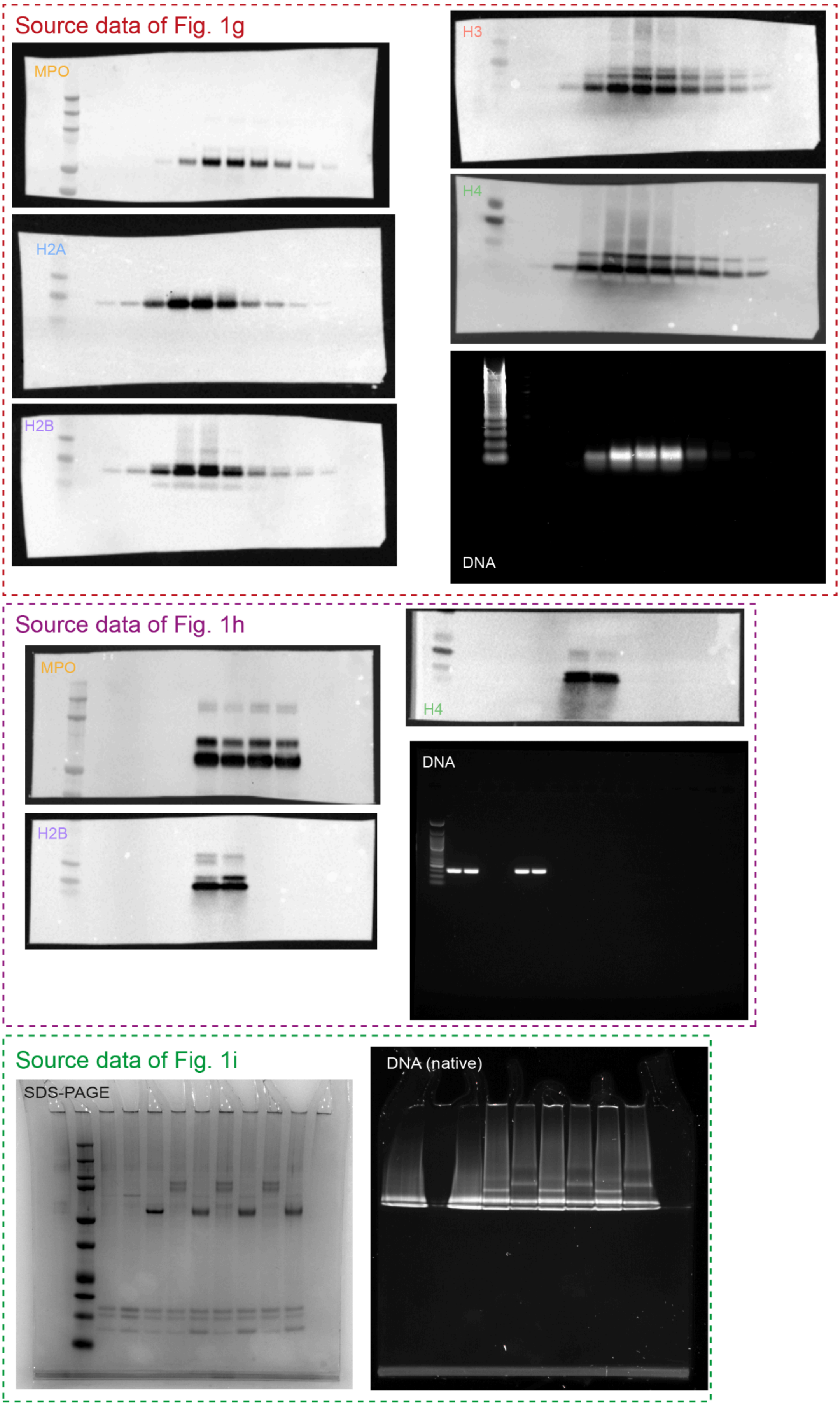

Supplementary Figure 22. Raw data of Fig. 1g,h,i.

Source data of Fig. 3a

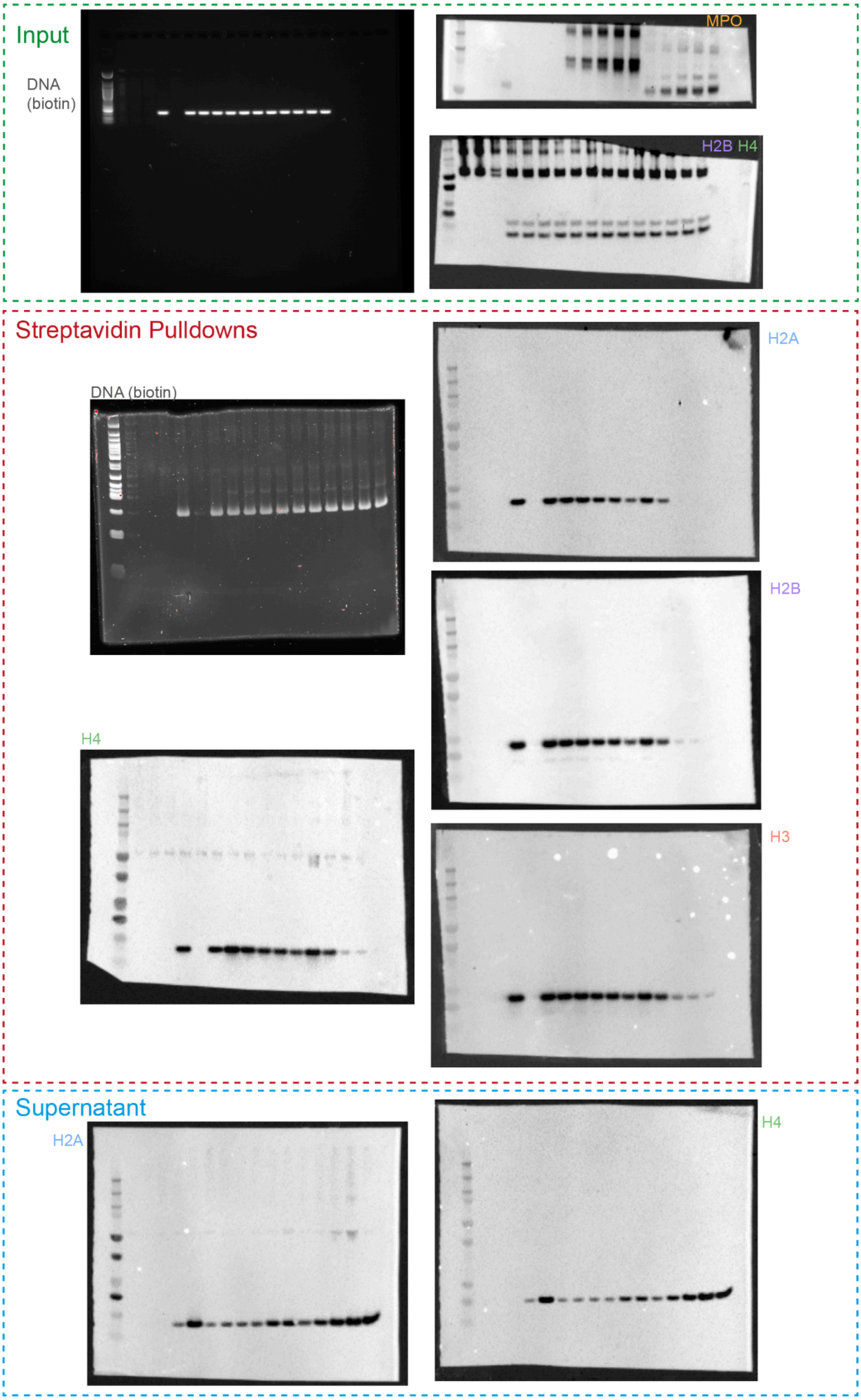

Supplementary Figure 23. Raw data of Fig. 3a.

Source data of Fig. 4d

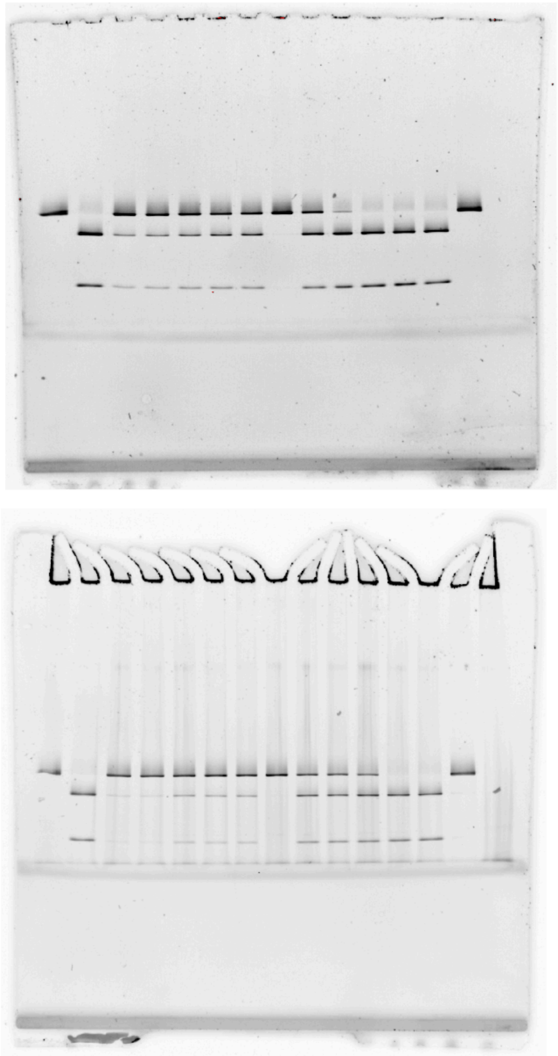

Source data of Fig. 4f

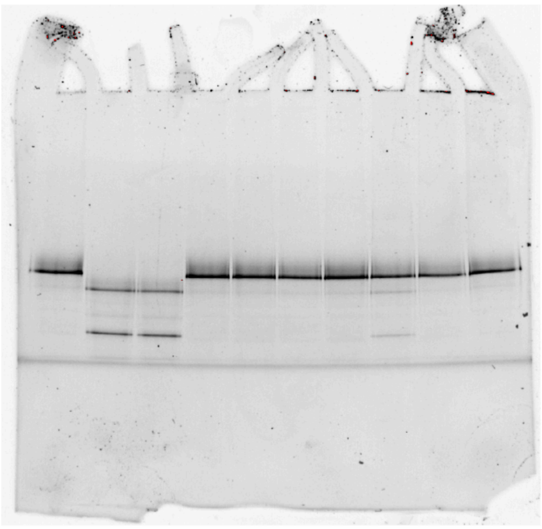

Supplementary Figure 24. Raw data of Fig. 4d,f.

Source data of Fig. 5c

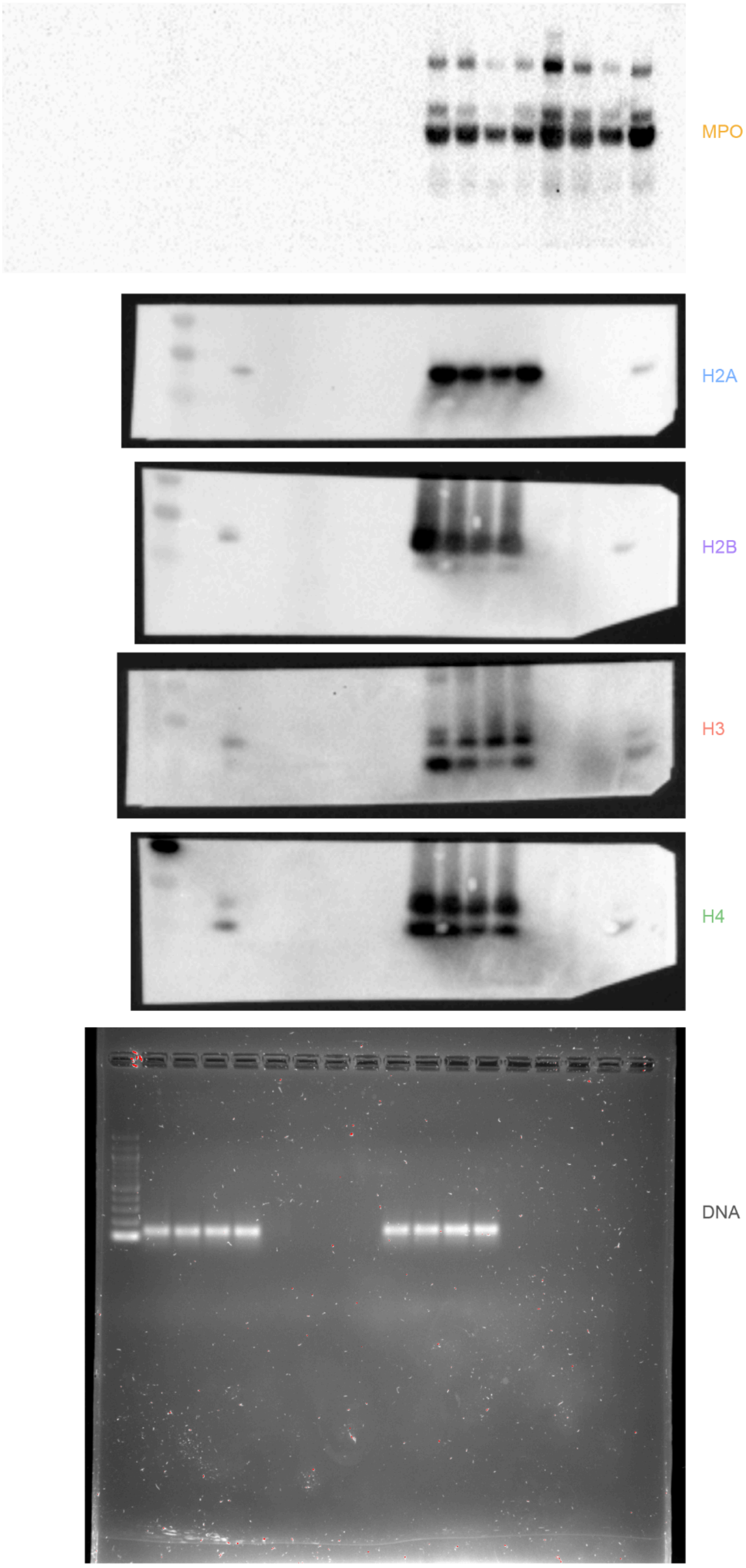

Supplementary Figure 25. Raw data of Fig. 5c.

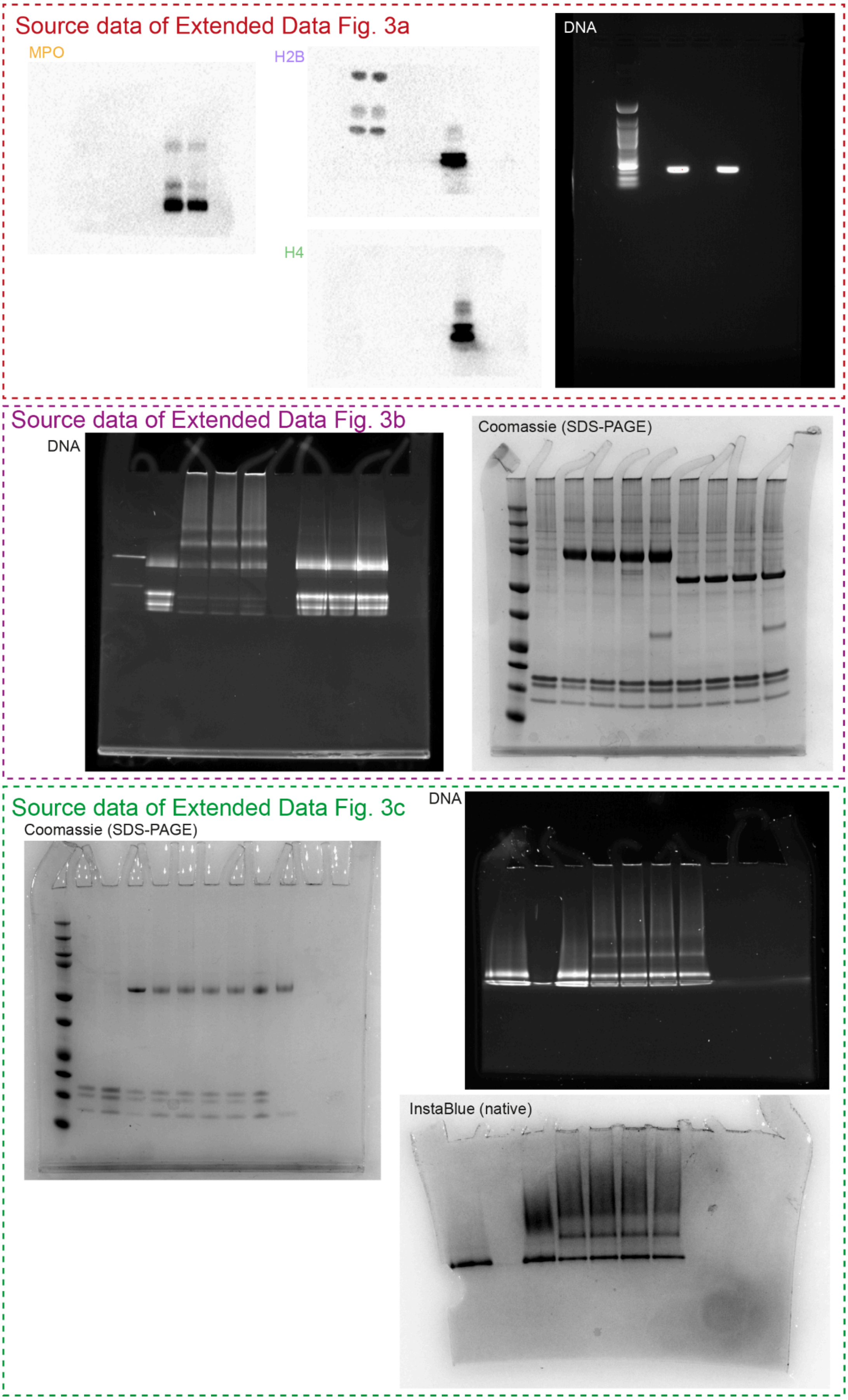

Supplementary Figure 26. Raw data of Extended Data Fig. 3a,b,c.

Source data of Extended Data Fig. 5e

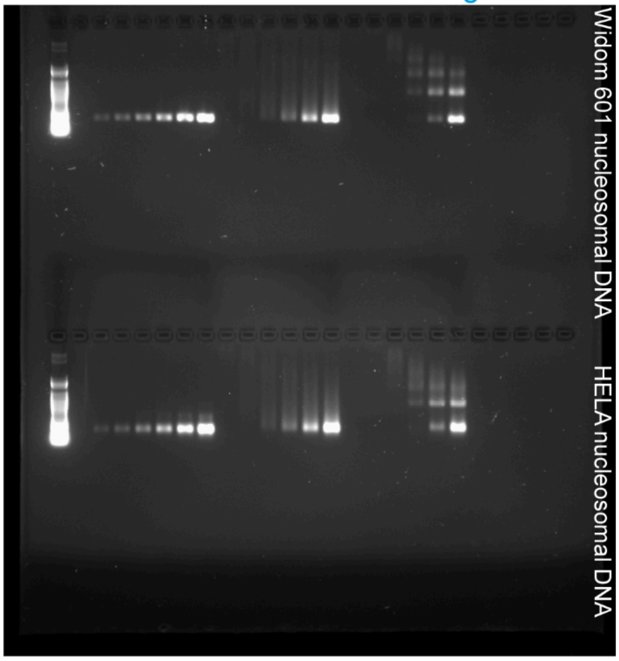

Source data of Extended Data Fig. 5c

Coomassie (SDS-PAGE)

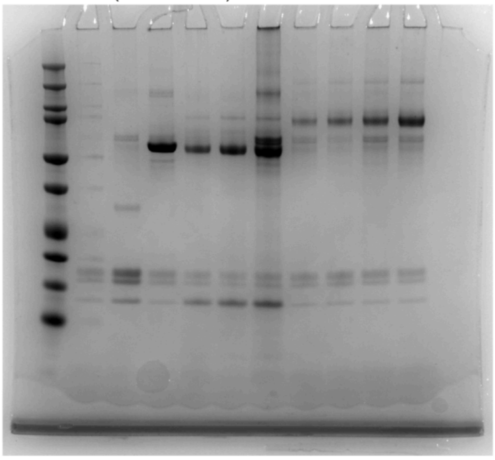

DNA (SDS-PAGE)

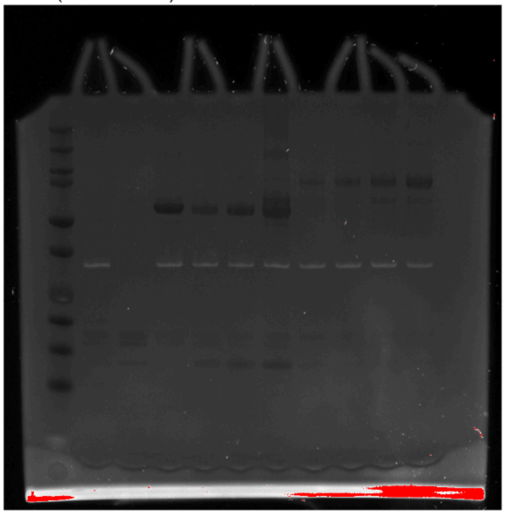

Silver stain (native PAGE)

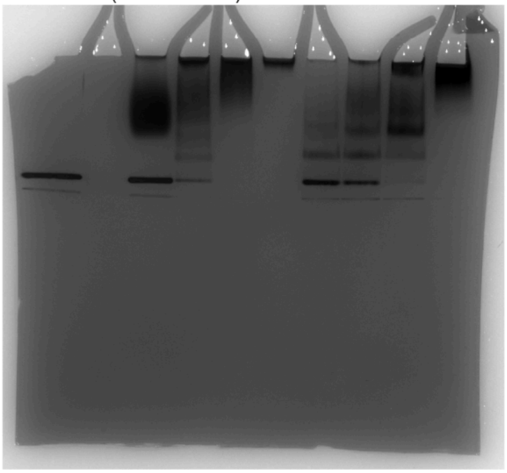

DNA (native PAGE)

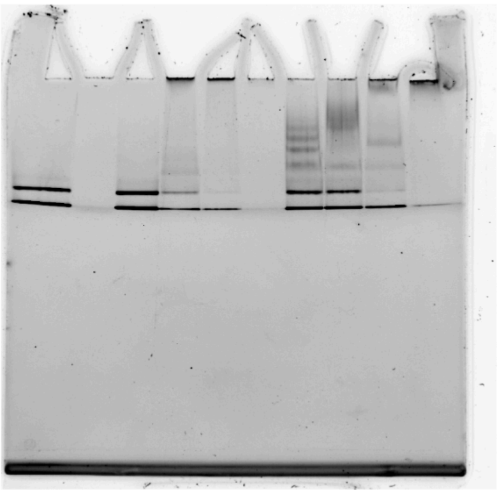

Supplementary Figure 27. Raw data of Extended Data Fig. 5c,e.

Source data of Extended Data Fig. 8c

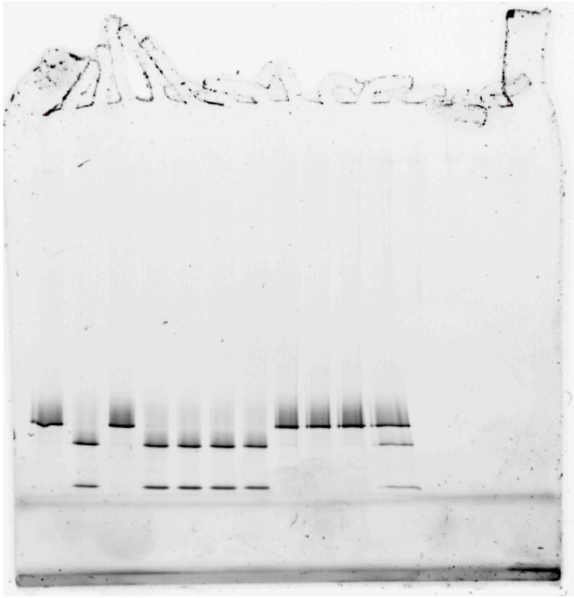

Source data of Extended Data Fig. 9b

MPO

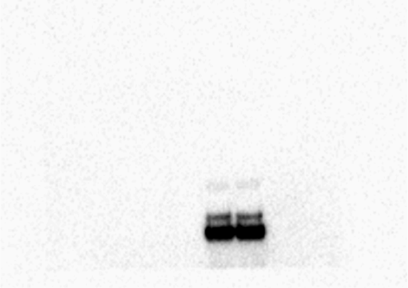

H2B

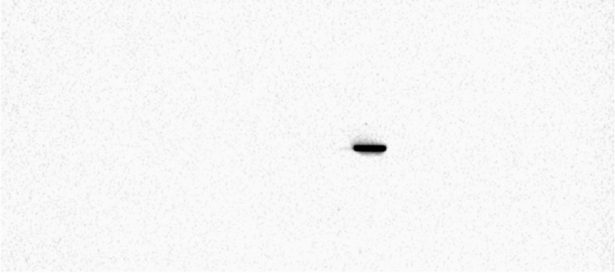

Source data of Extended Data Fig. 10b

3D9

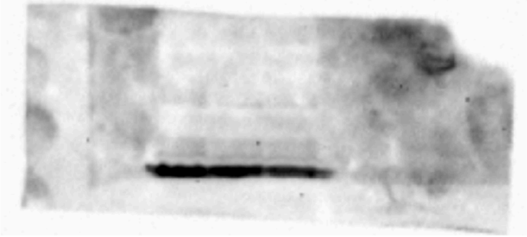

CitH3

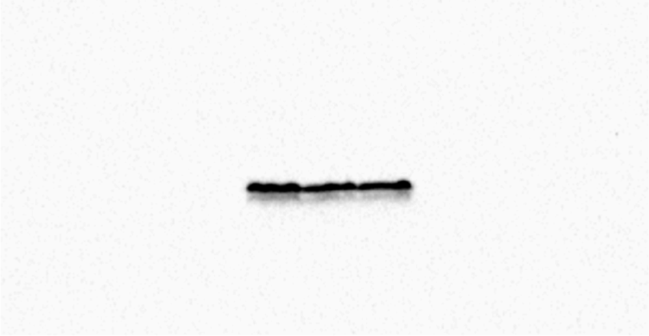

MPO

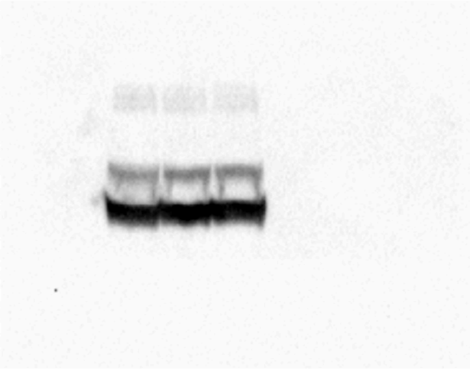

Supplementary Figure 28. Raw data of Extended Data Fig. 8c,9b,10b.

Source data of Supplementary Fig. 1c

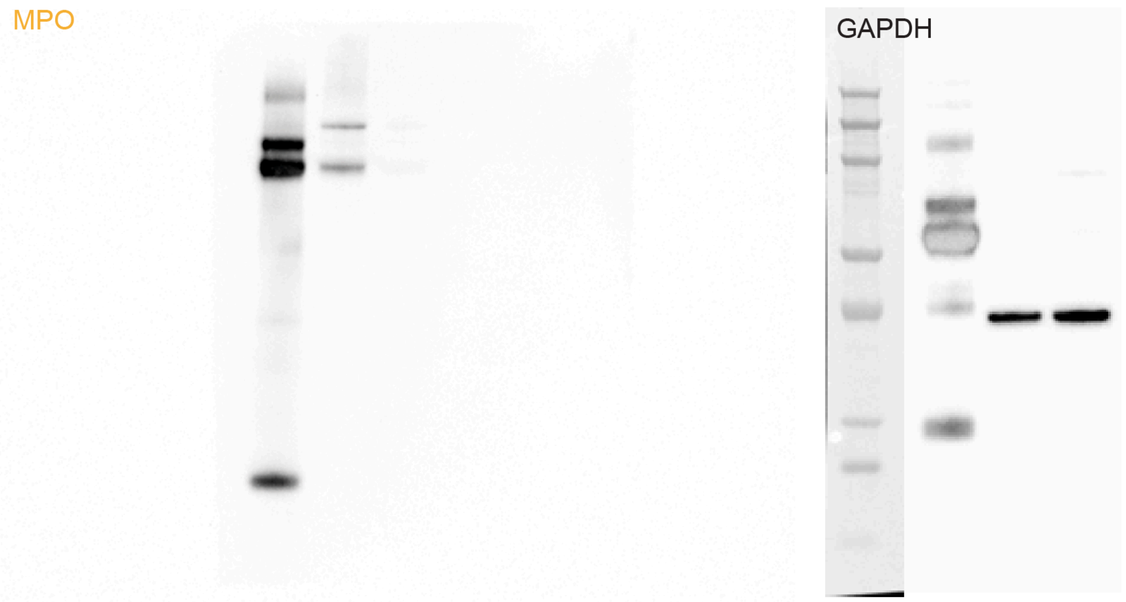

Source data of Supplementary Fig. 4b

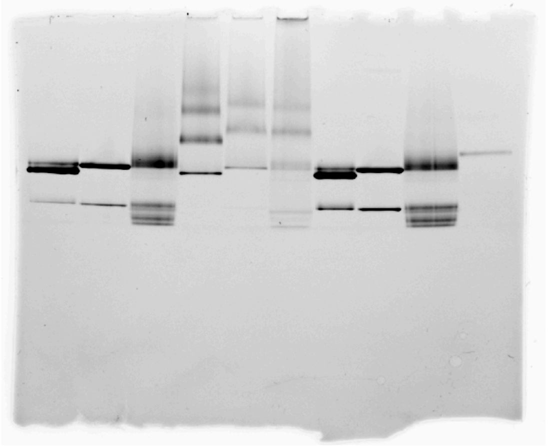

Supplementary Figure 29. Raw data of Supplementary Fig. 1c, 4b.
