# Supplementary material for: Myeloperoxidase transforms chromatin into neutrophil extracellular traps
Source: Nature. 2025 Sep 17;647(8090):747–56. doi: 10.1038/s41586-025-09523-9 (PMC12629992; doi:10.1038/s41586-025-09523-9)
Supplement: Supplementary file 2 — Reporting Summary [file 41586_2025_9523_MOESM2_ESM.pdf]

nature portfolio

Corresponding author(s): Stefan Raunser, Arturo Zychlinsky

Last updated by author(s): Jul 31, 2025

Reporting Summary

Nature Portfolio wishes to improve the reproducibility of the work that we publish. This form provides structure for consistency and transparency in reporting. For further information on Nature Portfolio policies, see our Editorial Policies and the Editorial Policy Checklist.

Statistics

For all statistical analyses, confirm that the following items are present in the figure legend, table legend, main text, or Methods section.

n/a	Confirmed	
☐	☒	The exact sample size (*n*) for each experimental group/condition, given as a discrete number and unit of measurement
☐	☒	A statement on whether measurements were taken from distinct samples or whether the same sample was measured repeatedly
☒	☐	The statistical test(s) used AND whether they are one- or two-sided<br>*Only common tests should be described solely by name; describe more complex techniques in the Methods section.*
☒	☐	A description of all covariates tested
☒	☐	A description of any assumptions or corrections, such as tests of normality and adjustment for multiple comparisons
☒	☐	A full description of the statistical parameters including central tendency (e.g. means) or other basic estimates (e.g. regression coefficient) AND variation (e.g. standard deviation) or associated estimates of uncertainty (e.g. confidence intervals)
☒	☐	For null hypothesis testing, the test statistic (e.g. *F*, *t*, *r*) with confidence intervals, effect sizes, degrees of freedom and *P* value noted<br>*Give P values as exact values whenever suitable.*
☒	☐	For Bayesian analysis, information on the choice of priors and Markov chain Monte Carlo settings
☒	☐	For hierarchical and complex designs, identification of the appropriate level for tests and full reporting of outcomes
☒	☐	Estimates of effect sizes (e.g. Cohen's *d*, Pearson's *r*), indicating how they were calculated

Our web collection on statistics for biologists contains articles on many of the points above.

Software and code

Policy information about availability of computer code

Data collection	EPU v2.7<br>EMMENU 4.0.9.55<br>AcquireMP v2024.2.0
Data analysis	CryoSPARC v4.3 and v4.4 (also CryoSPARC live)<br>Phenix v1.20 and v1.21<br>COOT v0.9.8.2<br>UCSF ChimeraX v1.8 and v1.9<br>Warp 1.0.9<br>TomoTwin 0.9.0<br>SPHIRE 1.5<br>IMOD 4.11<br>RELION 3.1.4<br>DiscoverMP v2024.2.0<br>https://github.com/ngimber/NanoNET

For manuscripts utilizing custom algorithms or software that are central to the research but not yet described in published literature, software must be made available to editors and reviewers. We strongly encourage code deposition in a community repository (e.g. GitHub). See the Nature Portfolio guidelines for submitting code & software for further information.

Data

Policy information about availability of data

All manuscripts must include a data availability statement. This statement should provide the following information, where applicable:

- Accession codes, unique identifiers, or web links for publicly available datasets
- A description of any restrictions on data availability
- For clinical datasets or third party data, please ensure that the statement adheres to our policy

The cryo-EM SPA data have been deposited at the EMDB and accompanying molecular models at the PDB under the following accession codes. rMPO/nucleosome complex: EMD-51295 (https://www.ebi.ac.uk/emdb/EMD-51295) and PDB 9GEN (https://doi.org/10.2210/pdb9GEN/pdb). Free nucleosome (5 minutes time point): EMD-51296 (https://www.ebi.ac.uk/emdb/EMD-51296) and PDB 9GEO (https://doi.org/10.2210/pdb9GEO/pdb). MPO monomer/nucleosome complex (5 minutes time point): EMD-51297 (https://www.ebi.ac.uk/emdb/EMD-51297) and PDB 9GEP (https://doi.org/10.2210/pdb9GEP/pdb). MPO dimer/nucleosome complex (5 minutes time point): EMD-51298 (https://www.ebi.ac.uk/emdb/EMD-51298; nucleosome focused map), EMD-51299 (https://www.ebi.ac.uk/emdb/EMD-51299; MPO focused map), EMD-51300 (https://www.ebi.ac.uk/emdb/EMD-51300; consensus map), EMD-51301 (https://www.ebi.ac.uk/emdb/EMD-51301; composite map) and PDB 9GEQ (https://doi.org/10.2210/pdb9GEQ/pdb). MPO dimer/nucleosome complex (intermediate state; 15 seconds time point): EMD-51302 (https://www.ebi.ac.uk/emdb/EMD-51302; nucleosome focused map), EMD-51303 (https://www.ebi.ac.uk/emdb/EMD-51303; MPO focused map), EMD-51304 (https://www.ebi.ac.uk/emdb/EMD-51304; consensus map) EMD-51305 (https://www.ebi.ac.uk/emdb/EMD-51305; composite map) and PDB 9GER (https://doi.org/10.2210/pdb9GER/pdb). MPO monomer/nucleosome complex (30 minutes plus SEC): EMD-51306 (https://www.ebi.ac.uk/emdb/EMD-51306). The cryo-EM data regarding the complexes with DTT-reduced native MPO were deposited under the following accession numbers: Nucleosome bound by one MPO monomer (2 minutes dataset): EMD-52865 (https://www.ebi.ac.uk/emdb/EMD-52865) and PDB 9IHD (https://doi.org/10.2210/pdb9IHD/pdb). Nucleosome bound by two MPO monomers (2 minutes dataset): EMD-52866 (https://www.ebi.ac.uk/emdb/EMD-52866) and PDB 9IHE (https://doi.org/10.2210/pdb9IHE/pdb). Nucleosome bound by one MPO monomer and one MPO dimer (5 minutes dataset): EMD-52867 (https://www.ebi.ac.uk/emdb/EMD-52867; consensus map), EMD-52868 (https://www.ebi.ac.uk/emdb/EMD-52868; nucleosome/MPO monomer focused map), EMD-52869 (https://www.ebi.ac.uk/emdb/EMD-52869; MPO dimer focused map), EMD-52870 (https://www.ebi.ac.uk/emdb/EMD-52870; composite map) and PDB 9IHF (https://doi.org/10.2210/pdb9IHF/pdb). The starting models for the model building can be found in the PDB under the following accession codes: 1MHL (https://doi.org/10.2210/pdb1MHL/pdb; native MPO dimer); 6AZP (https://doi.org/10.2210/pdb6AZP/pdb; recombinant MPO) and 6R1T (https://doi.org/10.2210/pdb6R1T/pdb; nucleosome). The utilized MatLab scripts for analysis of light microscopy data can be found in the GitHub repository (https://github.com/ngimber/NanoNET). All other data are available from the corresponding authors upon reasonable request.

Research involving human participants, their data, or biological material

Policy information about studies with human participants or human data. See also policy information about sex, gender (identity/presentation), and sexual orientation and race, ethnicity and racism.

Reporting on sex and gender	This information was not collected
Reporting on race, ethnicity, or other socially relevant groupings	N/A
Population characteristics	No data on population characteristics were collected
Recruitment	Anonymous blood donations from the Charité Campus Campus Mitte blood bank were purchased.<br>Sputum samples were collected by Dr. Stephanie Thee after explicit consent was obtained where patients could opt out.
Ethics oversight	Sample collection were approved by the ethics committee of Charité University Hospital, Berlin, Germany

Note that full information on the approval of the study protocol must also be provided in the manuscript.

Field-specific reporting

Please select the one below that is the best fit for your research. If you are not sure, read the appropriate sections before making your selection.

☒ Life sciences ☐ Behavioural & social sciences ☐ Ecological, evolutionary & environmental sciences

For a reference copy of the document with all sections, see nature.com/documents/nr-reporting-summary-flat.pdf

Life sciences study design

All studies must disclose on these points even when the disclosure is negative.

Sample size	The choice of sample size (at least three independent experiments) was based on common practice in the field.
Data exclusions	No data were excluded
Replication	All data were faithfully replicated by performing at least 3 independent experiments or more replications, as indicated in figure legends.

Randomization	This is not relevant to our study as experiments were performed in solo and therefore it was impossible to randomise the samples
Blinding	Blinding was not possible due to the fact that patient samples were collected when available on the day. All blood bank samples were anonymous.

Reporting for specific materials, systems and methods

We require information from authors about some types of materials, experimental systems and methods used in many studies. Here, indicate whether each material, system or method listed is relevant to your study. If you are not sure if a list item applies to your research, read the appropriate section before selecting a response.

Materials & experimental systems

n/a	Involved in the study	
☐	☒	Antibodies
☐	☒	Eukaryotic cell lines
☒	☐	Palaeontology and archaeology
☒	☐	Animals and other organisms
☒	☐	Clinical data
☒	☐	Dual use research of concern
☒	☐	Plants

Methods

n/a	Involved in the study	
☒	☐	ChIP-seq
☒	☐	Flow cytometry
☒	☐	MRI-based neuroimaging

Antibodies

Antibodies used	Dako rabbit anti-myeloperoxidase cat number A0398, lots 41244899/20051734/20030245<br>Cell signal mouse anti-H2A clone number L88A6 catalogue number 36365, lot 1<br>Abcam rabbit anti-H2B (ab1790) lot GR3267844-1<br>Abcam rabbit anti-H3 (ab1791) lot GR64776-1<br>Abcam rabbit anti-H4 (ab10158) lot GR3186358-1<br>anti-CitH3 (ab5103)<br>3D9 (in house generated)
Validation	The commercial anti-histone antibodies have been validated and quality tested by the manufacturer for the methods we have applied them to. Details can be found on their websites:<br><br>anti-H2A: https://www.cellsignal.com/products/primary-antibodies/histone-h2a-l88a6-mouse-mab/3636<br>anti-H2B: https://www.abcam.com/en-us/products/primary-antibodies/histone-h2b-antibody-chip-grade-ab1790<br>anti-H3: https://www.abcam.com/en-us/products/primary-antibodies/histone-h3-antibody-nuclear-marker-and-chip-grade-ab1791<br>anti-H4: https://www.abcam.com/en-us/products/primary-antibodies/histone-h4-antibody-chip-grade-ab10158<br>anti-CitH3: https://www.abcam.com/en-us/products/primary-antibodies/histone-h3-citrulline-r2-r8-r17-antibody-ab5103<br><br>The anti-MPO antibody was validated in neutrophil like PLB-985 MPO knockout cells.<br><br>3D9 and PL2.3 were validated in the following references (also cited in the manuscript):<br>Losman, M. J., Fasy, T. M., Novick, K. E. & Monestier, M. Monoclonal autoantibodies to subnucleosomes from a MRL/Mp(-)+/+ mouse. Oligoclonality of the antibody response and recognition of a determinant composed of histones H2A, H2B, and DNA. J. Immunol. 148, 1561–1569 (1992)<br>Dorothea Ogmore Tilley et al. Histone H3 clipping is a novel signature of human neutrophil extracellular traps eLife 11:e68283 (2022)

Eukaryotic cell lines

Policy information about cell lines and Sex and Gender in Research

Cell line source(s)	CVCL_2162 (PLB-985). Sex of cell, Female. Age at sampling, 36Y. Category, Cancer cell line. Provided by Mary Dinauer lab
Authentication	not authenticated, only used for antibody verification
Mycoplasma contamination	The PLB-985 cell line was used exclusively as a reference or control, and mycoplasma testing of the cell lines was not performed.
Commonly misidentified lines (See ICLAC register)	No commonly misidentified cell lines were used in this study

Plants

Seed stocks	N/A
Novel plant genotypes	N/A
Authentication	N/A
